# Homology models of the HIV-1 attachment inhibitor BMS-626529 bound to gp120 suggest a unique mechanism of action

**DOI:** 10.1002/prot.24726

**Published:** 2014-11-17

**Authors:** David R Langley, S Roy Kimura, Prasanna Sivaprakasam, Nannan Zhou, Ira Dicker, Brian McAuliffe, Tao Wang, John F Kadow, Nicholas A Meanwell, Mark Krystal

**Affiliations:** 1 Computer Assisted Drug Design, Bristol-Myers Squibb, Research and DevelopmentWallingford, Connecticut; 2 Department of Virology, Bristol-Myers Squibb, Research and DevelopmentWallingford, Connecticut; 3 Department of Chemistry, Bristol-Myers Squibb, Research and DevelopmentWallingford, Connecticut

**Keywords:** antiretroviral, molecular dynamics, protein modeling

## Abstract

HIV-1 gp120 undergoes multiple conformational changes both before and after binding to the host CD4 receptor. BMS-626529 is an attachment inhibitor (AI) in clinical development (administered as prodrug BMS-663068) that binds to HIV-1 gp120. To investigate the mechanism of action of this new class of antiretroviral compounds, we constructed homology models of unliganded HIV-1 gp120 (UNLIG), a pre-CD4 binding-intermediate conformation (pCD4), a CD4 bound-intermediate conformation (bCD4), and a CD4/co-receptor-bound gp120 (LIG) from a series of partial structures. We also describe a simple pathway illustrating the transition between these four states. Guided by the positions of BMS-626529 resistance substitutions and structure–activity relationship data for the AI series, putative binding sites for BMS-626529 were identified, supported by biochemical and biophysical data. BMS-626529 was docked into the UNLIG model and molecular dynamics simulations were used to demonstrate the thermodynamic stability of the different gp120 UNLIG/BMS-626529 models. We propose that BMS-626529 binds to the UNLIG conformation of gp120 within the structurally conserved outer domain, under the antiparallel β20–β21 sheet, and adjacent to the CD4 binding loop. Through this binding mode, BMS-626529 can inhibit both CD4-induced and CD4-independent formation of the “open state” four-stranded gp120 bridging sheet, and the subsequent formation and exposure of the chemokine co-receptor binding site. This unique mechanism of action prevents the initial interaction of HIV-1 with the host CD4+ T cell, and subsequent HIV-1 binding and entry. Our findings clarify the novel mechanism of BMS-626529, supporting its ongoing clinical development. Proteins 2015; 83:331–350. © 2014 Wiley Periodicals, Inc.

## INTRODUCTION

Antiretroviral drugs used to treat human immunodeficiency virus type 1 (HIV-1) infection can target several distinct steps within the viral lifecycle. Despite the range of antiretroviral agents available, there is a continued need for novel classes of drugs that target different stages of viral replication, primarily due to development of resistance to existing compounds, and a requirement for new agents to exhibit improved safety and tolerability profiles compared with currently available treatments.

Entry of HIV-1 into host cells occurs via a multistep process that requires interaction of the viral envelope spike with two cellular receptors in a sequential manner.^1^–^3^ The envelope spike is composed of trimers of the gp120 exterior glycoprotein that are noncovalently associated with the gp41 transmembrane glycoprotein; both proteins are formed by post-translational cleavage of the envelope protein gp160.^4^,^5^ Viral entry is initiated by interaction of gp120 with the N-terminus of the cellular CD4 receptor. This results in conformational changes to gp120 that assemble and expose a co-receptor binding site. Subsequent co-receptor binding results in further conformational changes that culminate in gp41-mediated membrane fusion.^4^,^5^ The gp120 protein is highly dynamic, undergoing multiple conformational changes in solution, making understanding of its structure and conformational dynamics challenging.^6^ However, crystal structures of gp120 in both the unliganded “closed” (UNLIG) and CD4/monoclonal antibody-bound (LIG) states show that the gp120 core consists of an inner and an outer domain connected by a four-stranded β-sheet bridging domain. The bridging domain is composed of two strands from the outer domain (β20–β21) and two from the inner domain (β2–β3).^7^–^11^ In the unliganded state,^11^ the bridging sheet strand order is β2–β3-β21–β20, where strands β2–β3 and β21–β20 form anti-parallel sheets while β3-β21 is a parallel β-sheet. This bridging sheet arrangement stabilizes the closed state and directs the V1/V2 loops toward the crown of the trimer spike, where it packs against the V3 loop and buries the co-receptor binding site. However, in the CD4-bound structures,^7^–^10^ CD4 binds at a highly conserved site, composed of regions from the outer and bridging sheet domains. The interaction of gp120 with CD4 stabilizes^7^,^12^,^13^ changes in the β21–β20 sheet conformation that leads to “opening” of the trimer spike and re-ordering of the bridging sheet (β3–β2-β21–β20). This directs the V1/V2 loops toward CD4 and away from V3 to form and expose the co-receptor binding site.^11^ As the gp120–CD4 interaction represents the first step in CD4-dependent HIV-1 cell entry, the conserved CD4 binding site provides an attractive target for new antiretroviral agents. Currently, there are no approved agents that target this initial gp120–CD4 interaction.

Several small-molecule attachment inhibitors^5^,^14^–^21^ (AIs) targeting the conserved CD4 binding region within gp120 have been described. A series of ketopiperazinamide-based small-molecule inhibitors are the most advanced.^5^,^14^–^18^ Optimization to improve the potency and pharmacokinetic profile of this class led to the discovery of BMS-626529.^22^–^24^ This molecule displays *in vitro* activity against HIV-1 envelopes with C-C chemokine receptor type 5 (CCR5-), C-X-C chemokine receptor type 4 (CXCR4), and dual tropism. It also is active against almost all HIV-1 subtypes tested except for subtype CRF01-AE and possibly group O.^24^ BMS-626529 is administered as a phosphonooxymethyl ester prodrug (BMS-663068), which was developed to improve the solubility and dissolution of BMS-626529.^22^ In an 8-day proof-of-concept study in treatment-naïve and -experienced HIV-1-infected subjects (all with subtype B infection), treatment with BMS-626529 (delivered as BMS-663068 in an extended-release formulation) resulted in substantial declines in plasma HIV-1 RNA (maximum median decrease from baseline ranged from 1.21 to 1.73 log_10_ copies/mL).^25^ Viral envelopes from the proof-of-concept study exhibited substantial inter- and intrasubject variability in susceptibility to BMS-626529. However, emergent resistance was not observed on population genotyping or phenotyping.^26^,^27^

Differing mechanisms of action are proposed for AIs; these are based on the observed activity against CD4-independent virus, direct inhibition of the virus–CD4 interaction,^14^,^16^ inhibition of CD4-induced changes in the gp120–gp41 structure,^28^ or by ordering of the unliganded “closed” trimer spike.^13^ Given the hypothesis that AIs interfere with the gp120–CD4 interaction, a potential resistance pathway for AIs is the emergence of CD4-independent virus; however, CD4-independent isolates are rarely isolated *in vivo*.^29^,^30^ Laboratory-derived envelopes with a CD4-independent phenotype (CXCR4- and CCR5-tropic) retain susceptibility to BMS-626529, while envelopes from viruses with substitutions associated with BMS-626529 resistance show no evidence of CD4-independent entry.^31^

Although structures representing various conformations of simian immunodeficiency virus and HIV-1 gp120 exist,^8^,^9^,^11^ there is currently no structural information available for BMS-626529 bound to gp120. In this article we describe structural models representing four different conformational states of HIV-1 gp120. The models were constructed utilizing known X-ray structures of HIV-1 gp120, and guided by published cryo-electron tomography,^32^,^33^ cryo-electron microscopy,^34^–^37^ and small-angle X-ray scattering (SAXS) data.^7^ These models were used for docking and molecular dynamics (MD) studies with BMS-626529 in order to determine plausible compound-binding pose(s), and provide insight into the mechanism of action of this class of antiretroviral compounds.

## MATERIALS AND METHODS

### Compounds

BMS-626529 and the earlier synthesized AIs (BMS-088, BMS-049, BMS-378806, BMS-488043)^15^,^17^,^38^–^40^ were prepared at Bristol-Myers Squibb.

### Construction of homology models of unliganded, intermediate, and liganded HIV-1 gp120 structures

The gp120 sequence from the HIV-1 JRFL strain (UniProt identifier: Q75760) was used for homology modeling, as it requires the least amino acid deletions or insertions within the published X-ray structures that were used as templates. However, in order to be consistent with the majority of published literature, the numbering of amino acids in gp120 within this study is based on the HIV-1 HXB2 reference strain sequence (UniProt identifier: P04578, residues 1–511). Residues 1–43 and 493–511 were not incorporated into the models to remove the dangling ends and reduce the size of the MD water box.

### Construction of a homology model for liganded HIV-1 gp120 (LIG)

**Figure 1:**
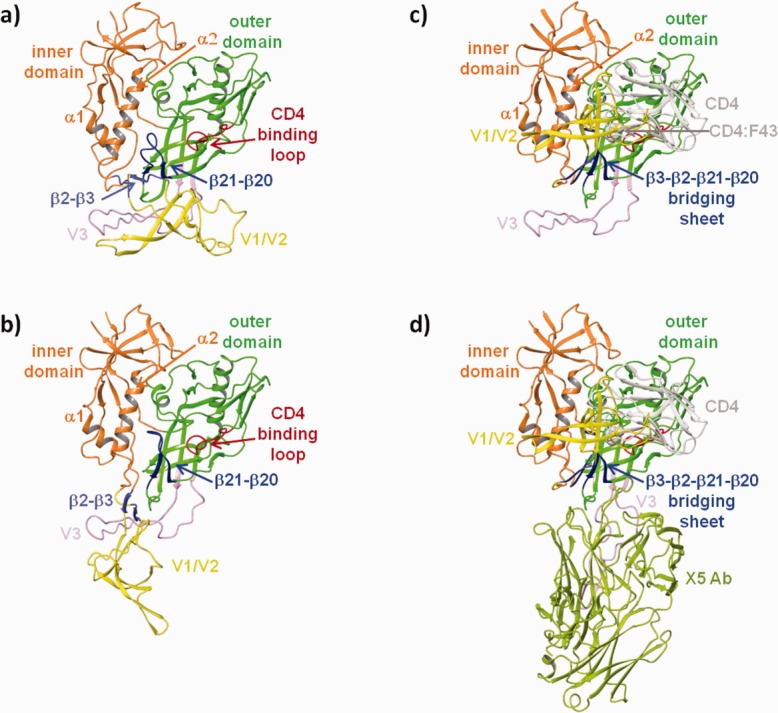
Homology models of the a) unliganded (UNLIG), b) pre-CD4 intermediate (pCD4), c) CD4-bound intermediate (bCD4), and d) liganded (LIG) conformations of HIV-1 gp120.

A variety of partial X-ray structures are available from the Protein Data Bank,^41^ representing multiple conformational states of gp120. In order to create a homology model of the liganded CD4-bound HIV-1 gp120 structure (LIG), three partial structures of HIV-1 gp120 (2B4C,^42^ 3JWD,^43^ and 3U4E^44^) were superimposed and merged into a single structure using the protein design package in QUANTA (QUANTA Modeling Environment, release 2006, Accelrys Software Inc., CA). The resulting structure was missing part of the V1/V2 loop (amino acids 171–188), which was constructed using the Prime Homology Modeling Package (Suite 2011, Prime version 3.0, Schrödinger LLC, NY).^45^ CD4 and the X5 antibody from the 2B4C structure were retained (Fig. 1d) to complete the model.

### Construction of a homology model for unliganded HIV-1 gp120 (UNLIG)

**Figure 2:**
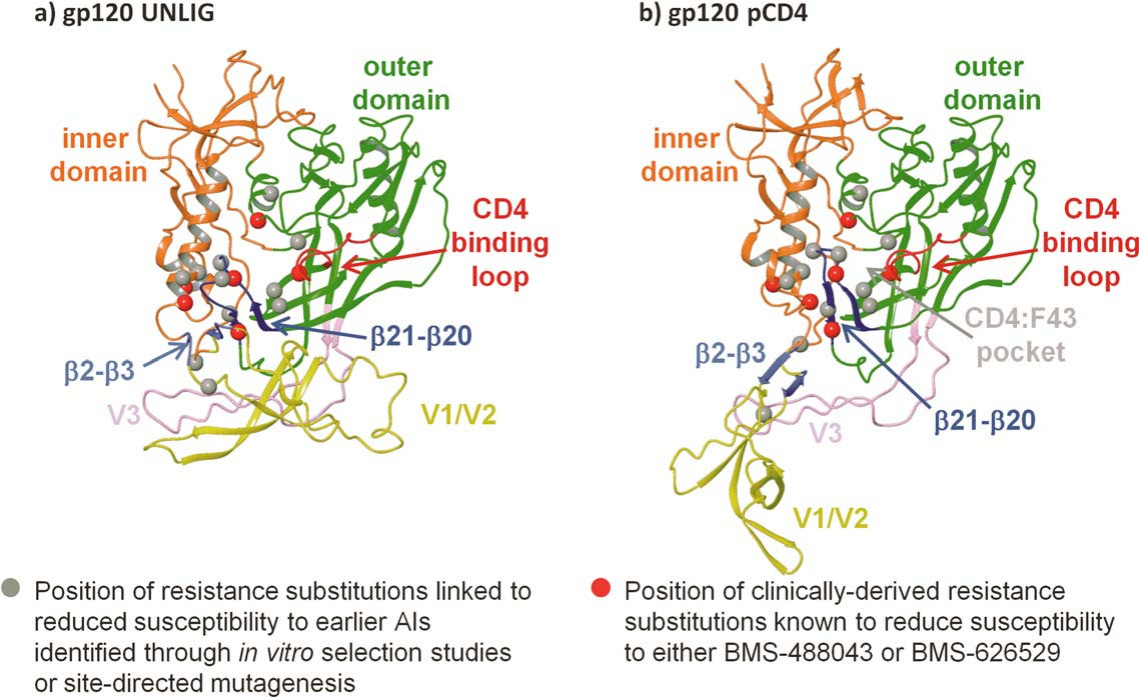
Position of attachment inhibitor resistance substitutions within homology models of a) unliganded gp120 (UNLIG), b) pre-CD4 binding intermediate (pCD4). Note: the models for the pCD4, bCD4, and LIG gp120 states all contain the HIV-1 gp120 water channel and CD4:F43 binding pocket so only one is depicted in the figure.

An HIV-1 core gp120 unliganded structure has been reported.^9^ However, it does not differ significantly from the CD4-liganded gp120 core structures.^8^ In contrast, the full-length unliganded “closed state” gp120 structure observed in the cleaved HIV-1 envelope trimer differs from the unliganded core structure primarily in the ordering and positioning of the bridging sheet domain.^11^ This is due to restraints imposed by the V1/V2 and V3 loops, which are not present in the monomer core structure. In the CD4-bound structures^7^–^10^ and the unliganded core structure,^9^ the bridging sheet (Fig. 1c–d) has the following order: β3–β2-β21–β20, and β3–β2, β2–β21, and β21–β20; all form anti-parallel β-sheets. In this arrangement, W427, from the β-turn of the β21–β20 sheet, binds deep into the liganded W427 binding pocket^46^ forming the gp120 water channel^47^ and the CD4:F43 binding pocket (Fig. 2b) and directs the V1/V2 loops toward CD4 and away from V3. This allows the formation and exposure of the co-receptor binding site.^7^,^11^,^13^ However, in the unliganded state,^11^ the bridging sheet strand order is: β2–β3-β21–β20. In this conformation, strands β2–β3 and β21–β20 form anti-parallel sheets, β3–β21 is a parallel β-sheet, and W427 is positioned near residues I109, S110, and D113 of α-helix-2 (α2) of the inner domain and the gp41 HR1 region^11^,^13^,^36^,^37^ that extends from the central cavity of the trimer envelope complex into the CD4 binding site. This bridging sheet arrangement directs the V1/V2 loops toward the crown of the trimer spike, where it packs against the V3 loop and buries the co-receptor binding site. It also opens up a large hydrophobic cavity between the outer domain and the bridging sheet.

In order to reduce the number of modeled amino acid side chains and loops, a homology model of the JRFL HIV-1 gp120 closed state was constructed from the HIV-1 gp120 crystal structures 2B4C^42^ and 4NCO.^11^ The structures were superimposed and 2B4C^42^ residues 253–296 and 331–418, 445–492, and 4NCO^11^ residues 44–252 (inner domain), 297–330 (V3 loop), 419–444 (β20–β21 loop) were merged into a single structure using the protein design package in QUANTA (refer above). The resulting structure was missing parts of the V1/V2 loop (amino acids 135–139, 150, and 179–190), which were constructed using the Prime Homology Modeling Package (refer above).^45^

### Construction of homology models for intermediate structures of HIV-1 gp120

To create a homology model of the CD4-bound intermediate (bCD4) state of HIV-1 gp120, the gp120 LIG structure was modified by replacing the V3 loop (residues 297–330) with the one from the 4NCO^11^ crystal structure. CD4 from the 2B4C^42^ structure was retained in the model. The gp120 pre-CD4 binding intermediate (pCD4) was constructed from the gp120 bCD4 by removing CD4 and adjusting the torsion angles of residues 116, 117, 202, and 203 to move the V1/V2 loops near the V3 loop in line with observations from SAXS.^7^

### Ligand docking

**Figure 3:**
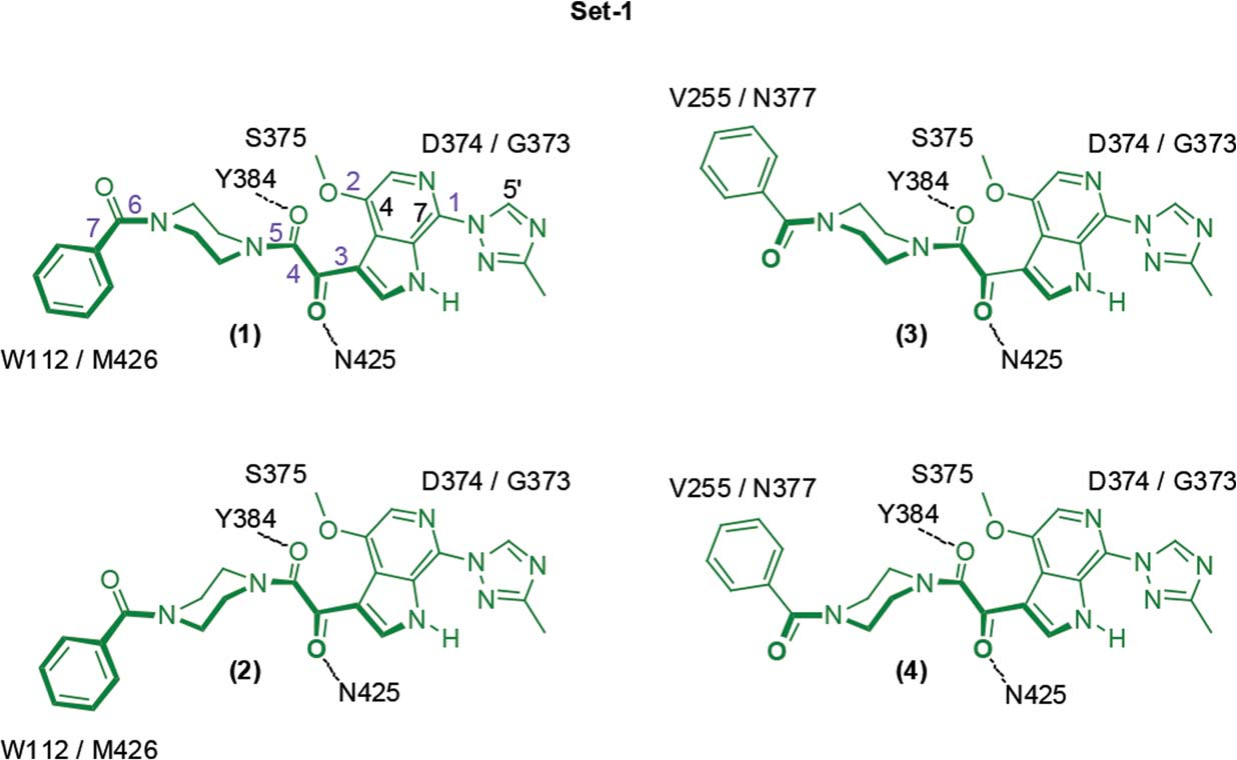
The four conformations of BMS-626529 docked into UNLIG gp120 from Set-1. Rotation of torsion-3 (purple) to the other minimum generates Set-2 and rotation of Set-1 and -2 about the long axis by ∼90 degrees generates Set-3 and -4, respectively. Key gp120 residues that are in close contact with the docked pose are shown. Dotted line depicts hydrogen bond.

BMS-626529 has seven rotatable bonds (Fig. 3:1) and the piperazine moiety can adopt a “chair,” or the higher energy “twist boat” and “boat” conformations. All rotatable bonds except 4 and 7 have minima at ∼0 and ∼180 degrees.^18^,^48^,^49^ However, due to steric and electronic constraints, the methoxy at C4 and 3-methyltriazole at C7 adopt deeper minima, with the methoxy pointing away from the oxoacetamide while the triazole hydrogen bonds with the azaindole NH group. The oxoacetamide moiety (torsion-4, O=C-C=O) has a minimum between ±87.4 and ±119.8 degrees as determined from 49 small-molecule X-ray structures from this series.^18^,^48^,^49^ While the piperazine ring can adopt several forms, only the “up” (Fig. 3:1 and 3) and “down” (Fig. 3:2 and 4) chair was used as starting conformations for the docking studies. The benzamide torsion-7 (Fig. 3:1) has minima between ±49 and ± 90 degrees, which were dependent on the piperazine ring conformation and crystal packing. This produced 16 unique conformations as starting points for docking studies.

Putative binding sites for BMS-626529 were identified following analysis of resistance mutation data, available AI structure–activity relationships (SARs),^50^,^51^ and biophysical and biochemical data, as described in the results and discussion section. Guided by these data, the 16 conformational forms of BMS-626529 were hand-docked and/or glide-docked^52^,^53^ into the gp120 water channel and CD4:F43 binding pocket that are present in the pCD4, bCD4 and LIG structures, and into the central hydrophobic cleft present within the HIV-1 gp120 UNLIG closed state. In addition, different side chain rotamers for amino acids W112, E370, N377, and N425 were used during the docking studies. For several of the glide docking studies into the UNLIG structure, hydrogen bond constraints with the Y384 side chain, and/or the backbone NH of N425, were given as options. The hydrogen bond constraints and side chain rotamers were selected based on MD refined hand-docked poses.

### Thrombin digestion

Protease susceptibility testing and preparation of soluble CD4 (sCD4) and HIV-1 JRFL gp120 were performed as previously described by Ho *et al*.^15^

### MD simulations

Each gp120 core/BMS-626529 complex was placed in a box of TIP3P^54^ water molecules with four sodium ions. The size of the box and the number of water molecules were determined by a minimum 9Å water layer covering the protein complex. The MD simulation was carried out with the Nanoscale Molecular Dynamics (NAMD) package.^55^,^56^ The Amber ff99SB-ILDN force field^57^,^58^ was used to model the protein, solvent and ions. A modified General Amber force field (GAFF)^59^ was used for BMS-626529 (see supplemental material). The Verlet integration and a 2/2/6 multitime step scheme^60^ were used and SHAKE^61^ was applied to all hydrogen bonds. The nonbonded interactions were tapered between 8 and 10 Å and a particle-mesh-Ewald (PME) method was used for long-range electrostatics.^59^,^62^,^63^ The simulation was run with periodic boundary conditions in an NPT ensemble at 300 K and 1 atm. The initial system was first relaxed with 250 steps of conjugate gradient minimization with the protein backbone constrained, followed by an additional 250 steps of unconstrained minimization. The MD simulation was heated in 10 K increments every 100 steps to 300 K (NVT ensemble) and equilibrated over 250 ps to the NPT ensemble. The MD production runs varied in length from 10 to 150 ns. Multiple simulations were run using different inhibitor docked poses. In some studies, selected constraints were used to test different hypotheses, and dihedral parameters for BMS-626529 were softened to enhance sampling. The simulations reported herein were performed without constraints.

### MD analysis

MD snapshots were collected every picosecond for data analysis. The stability of the MD for each model was monitored by root-mean-square deviation (RMSD) from the starting and mean structures using all heavy atoms of gp120 structure (amino acids 44–492). The RMSD for the inner domain utilized residues 44–119, 205–254, and 486–492, while the RMSD for the outer domain utilized residues 255–296, 331–362, 374–396, 413–421, 444–457, and 464–485; the variable loops, CD4 binding loop and β20–β21 sheet were not included. The program “R”^64^ was used to analyze and plot the RMSDs and computed distances. The 100-frame centered moving averages of the raw RMSD, and distance-time series were plotted in order to smooth out the high-frequency fluctuations and enable comparison between the RMSD and distance-time series. The mean closest heavy atom distances between BMS-626529 and key residues within the gp120 binding site were determined over the production phase of the simulation. For each 1 ps frame, the distance was calculated using the two closest heavy atoms (one from each residue), as described by Shrivastava *et al*.^46^

## RESULTS AND DISCUSSION

### Homology models of HIV-1 gp120

Homology models of the unliganded (UNLIG), pCD4, bCD4, and liganded gp120 (LIG) states of HIV-1 gp120 are shown in Figure 1a–d, respectively. In the UNLIG model (Fig. 1a), the V1/V2, V3 and bridging domain are in a nonCD4-binding conformations and there is a large hydrophobic cavity under the bridging domain and within the outer domain. In the gp120 pCD4 (Fig. 1b), the β20–β21 sheet moves toward the CD4 binding loop and into the hydrophobic cavity present in the outer domain of the UNLIG state placing W427 deep in the liganded W427 binding pocket,^46^ thereby forming the gp120 water channel and CD:F43 binding pocket. The rearrangement of the β20–β21 sheet disrupts the UNLIG bridging sheet domain between the β3–β21 strands, which destabilizes the trimer envelope and allows it to open, and the bridging sheet to rearrange to the CD4-bound state. In the CD4-bound model (bCD4, Fig. 1c), the β3–β2 sheet is zipped up against the CD4-stabilized β21–β20 sheet, forming the rearranged β3–β2-β21–β20 bridging sheet. The formation of the reordered bridging sheet moves V1/V2 close to CD4 domains 1 and 2 to fully expose the V3 loop and partially form the chemokine binding site. In the fully liganded gp120 (LIG, Fig. 1d) the V3 loop opens to complete the formation and exposure of the chemokine binding site, which is occupied by the X5 antibody in Figure 1d.

In the UNLIG model there is no hindrance to CD4 binding. However, the gp120 CD4:F43 binding pocket is only partially formed and CD4 is expected to bind weakly or not at all. Based on HDX^7^,^12^,^13^ and single-molecule fluorescence resonance energy transfer (smFRET) imaging studies,^3^ the gp120 UNLIG, pCD4, bCD4 states are in equilibrium. However, the gp120 UNLIG state is predominant due to stabilization by the tertiary and quaternary structure of the bridging sheet and V1/V2/V3 loops. However, CD4 binding shifts the equilibrium to the gp120 bCD4 state by trapping and stabilizing the β20–β21 sheet in the CD4 bound conformation. The CD4-stabilized rearrangement of the β20–β21 sheet, from the UNLIG (off position) to the bCD4 (on position), breaks the hydrogen bonding interactions between the β3–β21 strands, leading to the disruption of the UNLIG bridging sheet domain and the opening of the trimer envelope.

### Identification of putative BMS-626529 binding sites within HIV-1 gp120

#### Substitutions that affect susceptibility of HIV-1 to BMS-626529

In order to identify a putative binding site for BMS-626529, the amino acid positions of substitutions in gp120 linked to decreased susceptibility to AIs were mapped onto the gp120 UNLIG, pCD4, and bCD4 models (Fig. 2). These included substitutions shown to reduce susceptibility to BMS-626529 (S375, M426, M434, M475)^27^ and an earlier-generation AI, BMS-488043 (V68, L116, S375, M426).^65^ In addition, the amino acid positions of gp120 substitutions shown to reduce susceptibility to earlier-generation AIs in site-directed mutagenesis studies were also used in the analysis (W112, T257, F382, W427).^11^,^47^

The resistance substitutions predominantly mapped in and around the CD4 binding pocket and clustered in the structurally conserved hydrophobic cleft in the outer domain of the gp120 UNLIG model. In contrast, the same substitutions were clustered in and around the gp120 water channel and the CD4:F43 binding pocket in the gp120 pCD4, bCD4, and LIG models, suggesting two possible binding sites. The putative binding site within the hydrophobic cleft in the outer domain only appeared to be present in the gp120 UNLIG model, whereas the putative binding site within the gp120 water channel and CD4:F43 binding pocket only appeared to be present in the gp120 pCD4, bCD4 and LIG models.

#### Structure–activity relationship (SAR)

**Figure 4:**
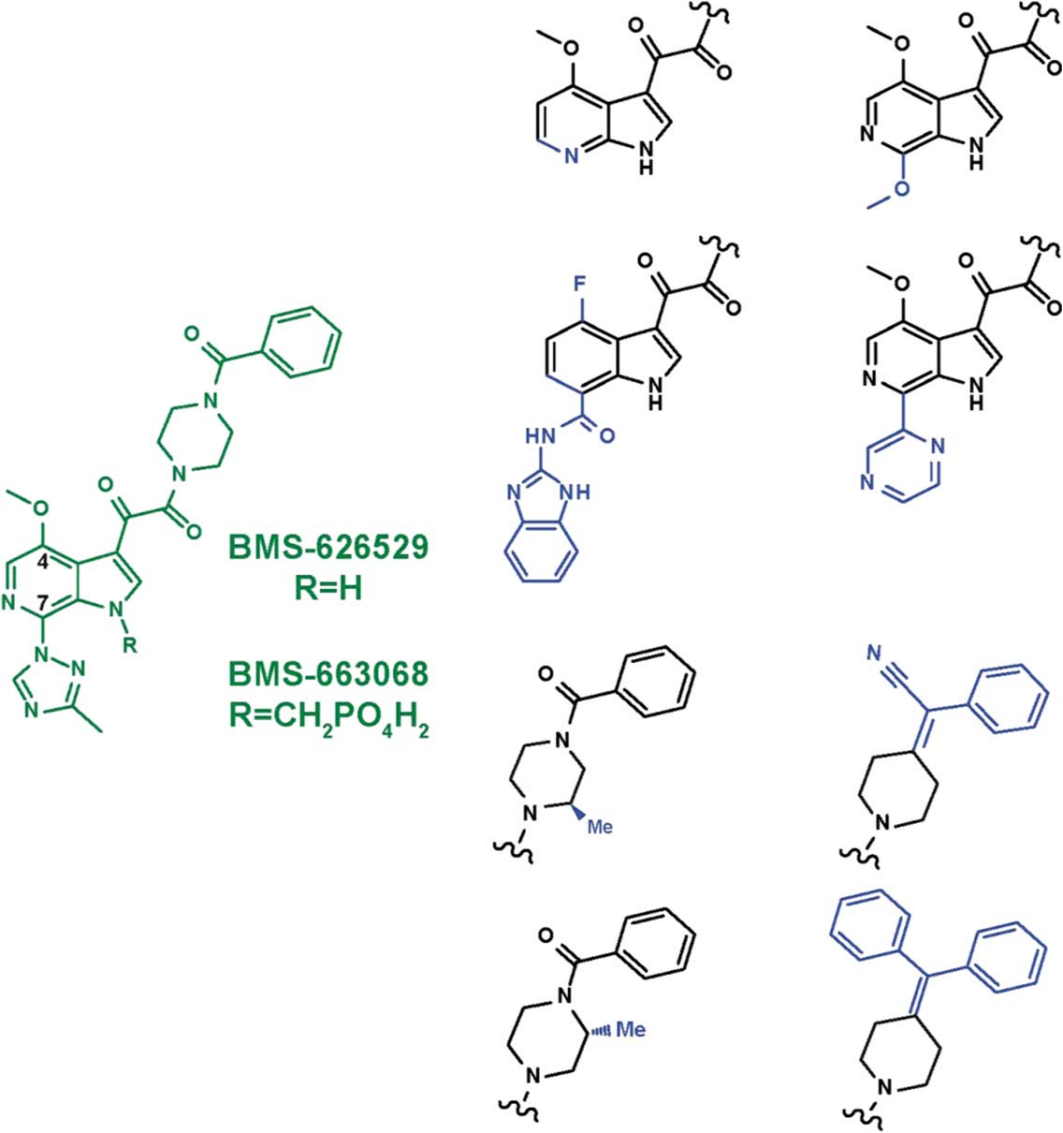
Structural diversity within the attachment inhibitor (AI) series. Blue regions highlight structural motifs that were used widely in earlier AIs that led to the development of BMS-626529 (shown in green).

SARs across the AI series were used to help identify the most plausible binding model. The BMS-626529 HIV-1 gp120 binding site must be able to accommodate a wide range of structural diversity within the AI series. Figure 4 shows structural motifs that were widely used in earlier AIs in the series that led to the identification of BMS-626529. The putative binding sites identified within the hydrophobic cleft in the outer domain of gp120 and the gp120 water channel/CD4:F43 binding pocket were compared against the full range of SARs for the AI series^17^,^18^,^38^,^49^,^52^,^53^,^66^,^67^ in order to determine their feasibility.

The proposed binding site for BMS-626529 within the gp120 water channel/CD4:F43 binding site of the gp120 LIG, pCD4, and bCD4 models did not accommodate the entire spectrum of structural motifs shown in Figure 4 (specifically, the 4-(diphenylmethylene) piperidine and the C7 2-amino-benzimidazole carboxamide moieties). In addition, the BMS-488043 C7-antibody conjugates described by Sato *et al*.^68^ and the 4′-methoxy indole attachment inhibitor with a C-7 furanyl PEG-DNP^69^ are not compatible with the model of AI binding within the gp120 water channel/CD4:F43 binding pocket in the gp120 LIG, pCD4, and bCD4 models. In contrast, the putative BMS-626529 binding site within the central hydrophobic cavity present in the gp120 UNLIG model was compatible with the entire range of observed SARs, suggesting that this model of compound binding has increased relevance.

#### Biochemical probing of binding site flexibility and binding mode

**Figure 5:**
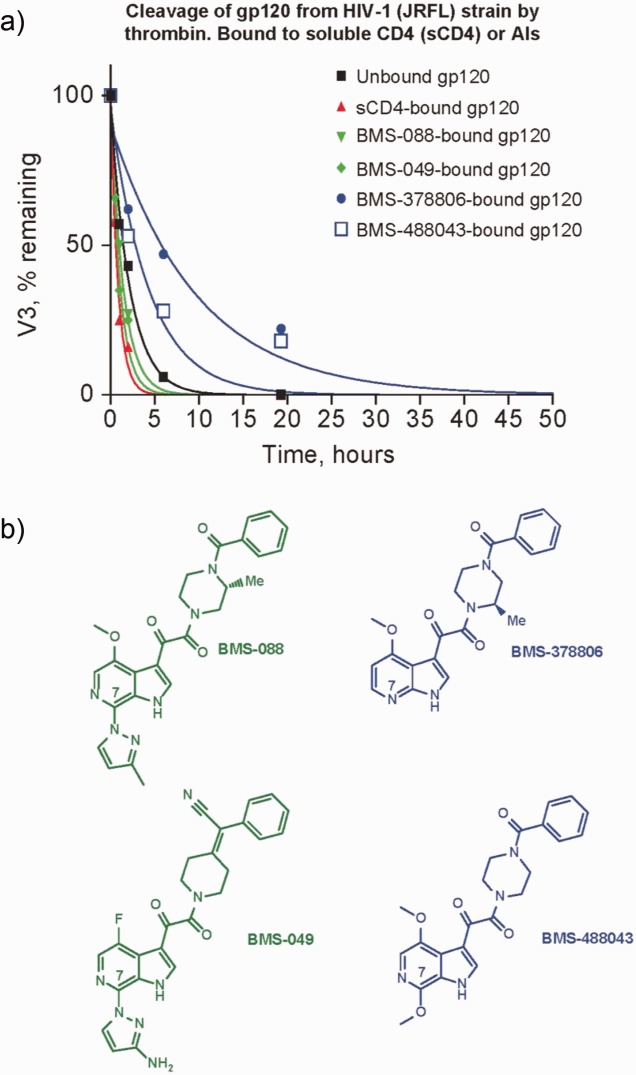
*a*) Effect of CD4 and attachment inhibitor (AI) binding on thrombin-mediated cleavage of the HIV-1 gp120 monomer; b) structures of the AIs.

Conformational changes in HIV-1 gp120 induced by AIs were measured through the ability of thrombin to cleave within the V3 loop of gp120.^15^ Thrombin-mediated cleavage of sCD4-bound gp120, unbound gp120, and gp120 bound to the AIs BMS-088, BMS-049, BMS-378806, and BMS-488043 was measured over a period of 50 h (Fig. 5a). Differences in susceptibility of the V3 loop to thrombin were seen between CD4-bound gp120, unbound gp120, and gp120 bound to the AIs. Relative rates of cleavage were as follows: CD4-liganded gp120 (16×) > BMS-049-bound gp120 (10×) > BMS-088-bound gp120 (7.1×) > unbound gp120 (4.5×) > BMS-488043-bound gp120 (2.5×) > BMS-378806-bound gp120 (1.0×). The rate of cleavage roughly correlated with the size of the moiety at the AI C7 position (Fig. 5b). This places directional constraints on the ligand binding mode, as C7 would need to be in a position to affect the V3 loop exposure, and at the same time, would need to be able to accept large substitutions up to the size of a linked antibody (in line with a recent study by Sato *et al*.).^68^ These data provide further evidence to support the proposed binding of BMS-626529 to the unliganded (gp120 UNLIG) conformation of gp120, and are not compatible with the proposed model of BMS-626529 binding within the gp120 water channel found in the pCD4, bCD4 and LIG conformations of gp120.

### Biophysical data

Further support for the model of AI binding to the gp120 UNLIG structure comes from studies by Si *et al*.^28^ and Myszka *et al*.^70^ Here, thermodynamic changes in HIV-1 gp120 were measured upon binding of an earlier AI, BMS-378806, or binding of sCD4. Interaction of BMS-378806 with gp120 was characterized by a binding enthalpy of –3.4 ± 0.5 kcal/mol and a binding entropy of 22 ± 2 cal/(K × mol),^28^ which contrasted with the extremely large enthalpy and entropy changes associated with binding of sCD4 to gp120, and the significant re-structuring of gp120 following sCD4 binding.^70^

The observed thermodynamic signature associated with binding of BMS-378806 supports a model in which the binding site is conserved across multiple states, which is in line with the presence of the structurally conserved hydrophobic cleft in the outer domain in the gp120 UNLIG state and exposed in the gp120 LIG state during an induced fit docking study by Parker *et al*.^69^ In contrast, binding of the AI within the gp120 water channel and CD4:F43 binding pocket in the gp120 LIG, pCD4 and bCD4 states would result in the restructuring of gp120. The large amount of gp120 restructuring that this binding model would require is inconsistent with the observed thermodynamic signature for AI binding.^28^ In addition, a recent study shows that BMS-378806 slows the rate of hydrogen deuterium exchange within gp120. It achieves this by binding with and stabilizing the closed trimer envelop complex.^13^ In contrast, NBD-556,^71^ which is a CD4 mimetic that binds within the gp120 water channel and CD4:F43 binding site, led to faster exchange.

### Docking of BMS-626529 into the gp120 UNLIG model

**Figure 6:**
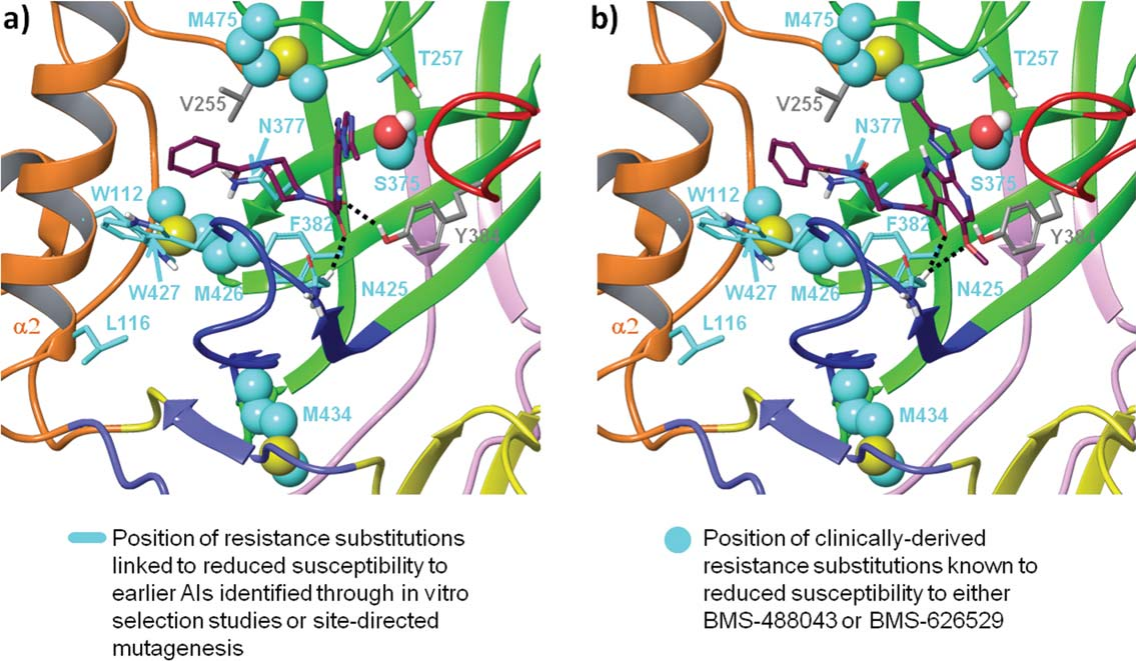
Models of BMS-626529 docked with HIV-1 gp120 in a) BMS-626529 Set-1:1 pose; b) Set-2:3 pose. Inner domain (orange), outer domain (green), V1/V2 (yellow), V3 (pink), β3–β2 sheet (light blue), β20–β21 (blue), CD4 binding loop (red), BMS-626529 (maroon). Black dotted lines depict hydrogen bonds.

**Figure 7:**
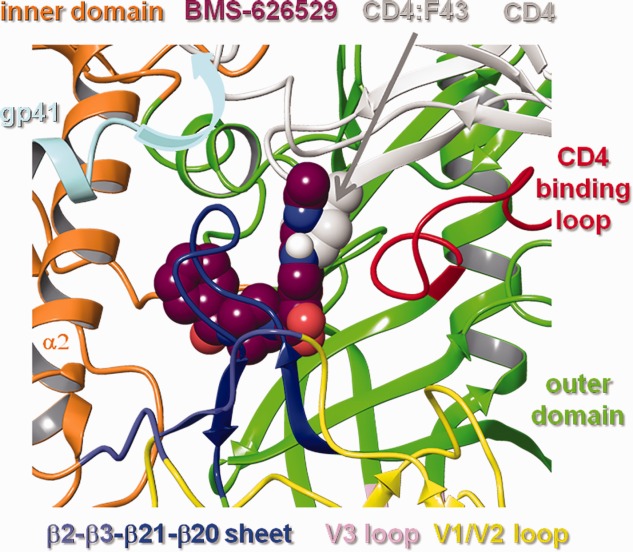
Models of BMS-626529 (Set-1:2 pose) docked with HIV-1 gp120 UNLIG. CD4 and gp41 are overlaid onto the structure. The 7-(3-methyltriazole) azaindole moiety occupies the space in the binding site adjacent to the CD4 binding loop required for CD4:F43 binding. The crystallographically unresolved region of the HR1 domain of gp41 (gray arrow) projects into the CD4 bind site near the β-turn of the β20–β21 sheet and adjacent to CD4.

BMS-626529 was docked into the central hydrophobic cavity present within the outer domain of the gp120 UNLIG model, as guided by AI SAR analysis, susceptibility studies, and biochemical data. When docked into the central cavity, BMS-626529 was surrounded by amino acid residues that represent the location of clinically- and laboratory-derived substitutions known to confer decreased susceptibility to BMS-626529 and/or the earlier AIs in the series (Fig. 6). In all of the docked models that gave a minimal fit to the criteria described above, the oxoacetamide dihedral angle (O=C-C=O torsion 4 (Fig. 3:1) is between –80 and –120 degrees. The substituted azaindole was positioned next to the CD4-binding loop to fill the space in the binding site required for CD4:F43 binding (Figs. 6 and 7), and the benzamide moiety was near α2 of the inner domain under the β20–β21 sheet and gp41. This general binding pose places the oxoacetamide moiety near the side chain of Y384 and the backbone NH of N425, allowing for hydrogen bonding interactions. However, binding pose sub-states are also readily accommodated. For example, the benzamide can be positioned near W112 and M426, while rotation of torsion angle-6 (Fig. 3:1) to the other minimum places it near V255 and N377. It should be noted that analogs containing the 4-(diphenylmethylene) piperidine moiety (Fig. 4) would simultaneously occupy both of these positions. Rotation of torsion-5 inverts the piperazine chair from an up- to a down-chair, and when combined with torsion-6 rotations, produces four unique binding poses. This group can be expanded to eight binding poses by simply rotating torsion-3 between its two minima. In the first set of four poses (Set-1), the AI is docked with the C4-methoxy pointing toward the piperazine ring and the C4-methoxy group is buried deep in the hydrophobic cleft, positioned near T257 and S375. In addition, the azaindole C5, N6, and the triazole C5′ positions occupy a position near G473, D474, and M475. This explains why substitution at these positions results in reduced activity.^49^,^52^ Rotation of torsion-3 to the other minimum generates a second set (Set-2) of four poses and points the azaindole out of the pocket, through the space between the β20–β21 sheet and the CD4-binding loop. In this orientation of the azaindole, C5, N6 and triazole C5′ positions project into the surrounding solvent and may represent a lower affinity-binding mode that is populated when AIs are modified at these positions. Two more sets (Set-3 and Set-4) of four poses each can be obtained by simply rotating the poses in Set-1 and Set-2, respectively, about the AI long axis by ∼90 degrees. This rotation moves the azaindole group into the slot between the CD4-binding loop and the β20–β21 sheet, but like Set-2, the poses of Set-3 and Set-4 expose the azaindole C5, N6, and triazole C5′ positions to the solvent, and provide poor explanations of the SAR. The hand docking exercise produced 16 viable poses, but only the four poses in Set-1 strongly fit with the AI SAR data. Glide docking, with hydrogen bond constraints, identified the four poses from Set-1 and one pose from Set-2.

BMS-626529 was also docked into the gp120 water channel and CD4:F43 binding pocket within the gp120 LIG, bCD4, and pCD4 models (data not shown). As expected, docking into this channel with the benzamide moiety pointing either toward or away from the CD4 binding site (as proposed in previous models of AI binding^47^,^72^,^73^ failed to produce a model that could accommodate the full scope of the AI SAR. Specifically, the 4-(diphenylmethylene) piperidine moiety (Fig. 4) does not fit in the channel when facing away from the CD4 binding site. Further, large groups at the 7-position, such as the amino-benzimidazole amide moiety (Fig. 4), cannot extend beyond the glycosylation sites at N262 and N448 when the structure is inverted. Despite these restrictions, some members of the AI series, such as BMS-378806 and BMS-488043, could be docked into the water channel present in the gp120 pCD4, bCD4, and LIG models. While we cannot rule out the possibility of multiple binding modes for different members of the AI compound collection, a unified model that binds the majority of compounds from this congeneric series seems more likely. Thus, only models of BMS-626529 bound to the UNLIG conformations of gp120 were taken forward for evaluation and analysis in MD simulation studies.

### MD simulations and refinement of docked poses

Multimicrosecond (μs)-long MD simulations show that compounds can spontaneously and correctly dock into some protein families.^74^ In these studies, the compound moves around in the solvent where it samples a large set of conformational and solvation states until it finds and enters the binding vestibule. From the binding vestibule, the ligand can either re-enter the solvent or sample with the protein target a smaller subset of conformational and solvation states until it enters the binding site. Once in the binding site, the protein, ligand, and environment continue to sample smaller and smaller states as the complex settles into the bound state or an ensemble of bound sub-states. The goal of the docking study presented here is to accelerate the ligand entry into the binding site and let the MD refine the binding pose(s). Thus, multiple MD simulations on the 25–150 ns timescale were conducted to test the dynamic stability of the BMS-626529-docked models and examine the interactions between BMS-626529 and residues at positions within gp120 associated with substitutions known to reduce susceptibility to AIs. These simulations were performed in explicit solvent, using the gp120 UNLIG/BMS-626529 docked poses discussed above as starting structures.

As expected from the results of earlier structure^8^,^9^,^11^,^32^–^37^ and HDX studies,^7^,^12^,^13^ the MD mean gp120 structures differed from simulation to simulation as larger fluctuations were observed in the inner domain, the variable loop, segments of the CD4-binding loop, and the β20–β21 sheet. Two starting poses from Set-1 converged over the course of the simulation to a common dynamic AI binding pose, with the caveat that the protein from each simulation had structural differences. One pose from Set-2 settled into a stable simulation. The remaining binding poses either partially exited the gp120 binding site (re-entered the binding vestibule), or induced large structural distortions in gp120 and were discarded as potential binding models. The following will focus on the two models from Set-1 that strongly fit with the SAR for this AI series and the one model from Set-2 that stabilized.

### Accelerated sampling of gp120 UNLIG/BMS-626529 Set-1 simulation

To further accelerate the ligand sampling within the binding site, MD simulations were carried out with soft torsion parameters on the four bound poses from Set-1. The goal of this study was to determine if the benzamide has a preferred location within the binding site, and to determine if there is room within the binding site for BMS-626529 to rearrange to the preferred conformation. In the two models where the phenyl ring of the benzamide moiety was initially docked near V255 and N377, the amide flipped to move it near W112 and M426 between 16 and 19 ns (down) or 37 and 39 ns (up), depending on the initial piperazine ring conformation. In both simulations, the amide flip occurred through a piperazine twist-boat conformation and pyramidal benzamide nitrogen. The phenyl ring moved through an arch from a position near V255 and N377, past F210 and F382, to end in a position close to W112 and M426. Only minimal movement was observed in the two simulations that started with the phenyl group of the benzamide moiety near W112 and M426. Once equilibrated, all four simulations remained semistable throughout the remainder of the 100-plus-nanosecond simulations, with the piperazine ring flipping from chair-to-chair, and the oxoacetamide moiety hydrogen bonding with the side chain of Y384 and backbone NH of N425 and occasionally K421. The two structures with the benzamide starting near W112 and M426 show tight convergence, while the other two differed in the pucker of the piperazine ring and placement of the benzamide group. All four structures differ in the protein structures as larger fluctuations were observed in layer 1 of the inner domain, all of the variable loop, portions of the CD4-binding loop, and the β-turn residues of the β20–β21 sheet (Supporting Information Fig. S1). The differences observed in the AI conformations of the MD mean structures (Supporting Information Fig. S1) were attributed to differences in protein conformations, soft AI parameters and incomplete sampling. While the MD simulations identified a preferred orientation for the benzamide moiety, they also highlight the accessible room within the binding site required to accommodate the 4-(diphenylmethylene) piperidine and 4-(phenylacrylonitrile) piperidine moieties (Fig. 4).

### gp120 UNLIG/BMS-626529 Set-1:1, Set-1:2 and Set-2:3 simulations

**Figure 8:**
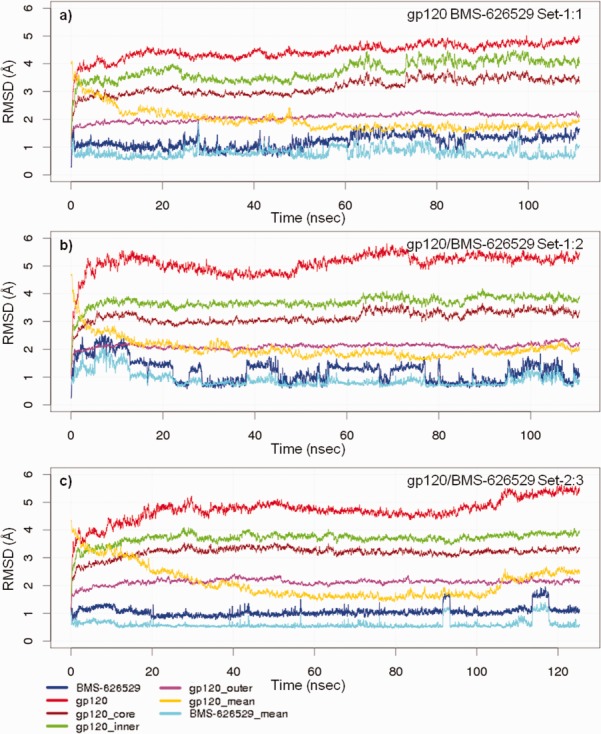
Root-mean-square deviation (RMSD) between the simulation frames and the starting coordinates or molecular dynamics (MD) mean coordinates. MD simulation a) gp120 UNLIG/BMS-626529 Set-1:1 binding pose, b) gp120 pCD4/BMS-626529 Set-1:2 binding pose, and c) gp120 pCD4/BMS-626529 Set-2:3 binding pose.

The RMSD between the simulation frames and the starting coordinates/MD mean coordinates for the gp120 UNLIG/BMS-626529 simulations starting from docked poses of Set-1:1 (Fig. 6a), Set-1:2 (Fig. 7) and Set-2:3 (Fig. 6b and Fig. 3:3) with rotation of torsion-3 to the other minimum) are illustrated in Figure 8. In all three simulations, the structure drifted away from the initial model (Fig. 8a–c, red) over the first 15 to 20 ns, where it stabilized and fluctuated about the MD mean conformation (Fig. 8a–c, yellow). A large portion of the drift that occurred in the first 15 ns was due to the rearrangement of the inner domain, the variable loop, segments of the CD4-binding loop, and the β-turn residues of the β20–β21 sheet, as the structure moved away from the conformation observed in the trimer spike complex. The large fluctuations observed in these regions of HIV-1 gp120 are in agreement with results from earlier structure^8^,^9^,^11^,^32^–^37^ and HDX studies.^7^,^12^,^13^ Even with the variable loops, segments of the CD4-binding loop, and the β20–β21 sheet excluded from the RMSD analysis, a large drift was observed for the HIV gp120 core (Fig. 8a–c, green). The largest contributor to the drift is the inner domain (Fig. 8a–c, brown), while the structurally conserved outer domain showed minimal drift and stabilized within the first 5 ns of the simulations (Fig. 8a–c, pink). Similar to the accelerated simulations, the MD mean gp120 structures differ from simulation to simulation, due mostly to the larger fluctuations observed in the inner domain, the variable loop, segments of the CD4-binding loop and the β-turn residues of the β20–β21sheet. The key difference between the three runs is associated with the conformation of BMS-626529. In the Set-1:1 and 2 simulations, the piperazine ring is in different chair configurations, which results in a slightly different placement of the benzamide group.

In the Set-1:1 simulation (Fig. 6a and Fig. 3:1), the up-chair conformation stabilized within the first nanosecond. BMS-626529 fluctuated about the starting conformation, which is only slightly offset from the MD mean conformation (Fig. 8a, blue, light blue) throughout the remainder of the simulation, with occasional sampling of the twist-boat and down-chair conformation.

In the Set-1:2 simulation (Fig. 7 and Fig. 3:2), the AI piperazine ring started in the down-chair conformation. In this simulation, the AI took about 15 ns to equilibrate and become semistable. For the remainder of the simulation, BMS-626529 fluctuated about the starting and MD mean conformation (Fig. 8b, blue, light blue) with the piperazine ring continually flipping between the up-chair, twist-boat and down-chair conformation (Fig. 8b, blue). Despite lower overall drift in the protein and inhibitor observed in the Set-1:1 simulation, a notable kink slowly developed in α2 of the inner domain over the first 20 ns that rapidly vanished between 24 and 28 ns. Once equilibrated, the two Set-1:1 and 2 simulations showed tight convergence with respect to the HIV-1 gp120 and BMS-626529 interactions. The AI oxoacetamide moiety forms nearly continuous hydrogen bonds with the side chain of Y384 and backbone NH of N425 and occasionally the side chain K421. This occurred while the phenyl group of the benzamide moiety fluctuated near I108, I109, and W112 from α2 of the inner domain and M426 from the β20–β21 sheet. In the Set-2:3 simulation (Fig. 6b and Fig. 3:3) with rotation of torsion-3 to the other minimum), the starting AI docked pose placed the phenyl ring of the benzamide near V255 and N377, the piperazine ring is in the up-chair, and the azaindole is docked between the β20–β21 sheet and the CD4 binding loop. BMS-626529 took about 20 ns to equilibrate and stabilize. During the simulation it only sampled the down-chair conformation two times (Fig. 8c, blue, light blue). During the equilibration period, the β-turn residues of the β20–β21 loop moved over the inhibitor where the following side chain-to-backbone hydrogen bond pairs stabilized it: W427-Q105, Q428-G473, or M475, and benzamide carbonyl W475. Once the complex equilibrated, it remained stable for the remainder of the 100-plus-nanosecond simulation.

In all of the simulations, the MD mean protein structures differ as larger fluctuations were observed in the inner domain, the variable loop, and portions of the CD4-binding loop and the β-turn residues of the β20–β21 sheet (Supporting Information Figs. S1 and S2). Videos of the last 10 ns of the Set-1:1, Set-1:2, and Set-2:3 simulations are provided in supporting material S3, S4, and S5, respectively.

### gp120 UNLIG/BMS-626529 Set-1:3 and Set-1:4 simulations

The Set-1:3 and 4 simulations (Figs. 3:3 and 4), where the phenyl ring of the benzamide group was docked near V255 and N377, only remained stable for a few nanoseconds. BMS-626529 slowly worked partially out of the binding site as water molecules move in and under the AI azaindole and oxoacetamide moieties to break the hydrogen bonds between the side chain of Y384 and backbone NH of N425. The binding pose with the down chair conformation (Fig. 3:4) remained semistable for ∼50 ns, but left the binding site with a similar path as the up chair pose (Fig. 3:3). Both molecules rotated as a unit, with the azaindole ring moving between the CD4-binding loop and the residues 470–473, while the phenyl group of the benzamide moiety moved near W112 and M426. For the remainder of the 100-plus-nanosecond simulations, the AI benzamide moiety remained anchored near W112 and M426, while the azaindole/triazole moieties floated in the pocket near the CD4 binding loop and residues 470–473.

### BMS-626529/gp120 interactions

Throughout the gp120 UNLIG/BMS-626529 MD simulations, BMS-626529 remained firmly seated in the central hydrophobic cavity of gp120 and under the β20–β21 sheet, blocking it from folding into the CD4-binding conformation and forming the four-stranded (β3–β2–β21–β20) bridging sheet that is required for formation and exposure of the chemokine receptor binding site. Consistent with the favorable entropy observed with gp120/BMS-378806 binding,^28^ these models suggest an adaptable binding mode that persists within multiple substates of UNLIG gp120.

Throughout the course of both the accelerated and normal MD simulations, BMS-626529 made close contact with a range of amino acid residues associated with both clinical and laboratory-generated substitutions known to decrease susceptibility to AIs. This was despite the high level of conformational sampling of the β-turn residues of the β21–β20 sheet that persisted throughout all of the simulations.

**Figure 9:**
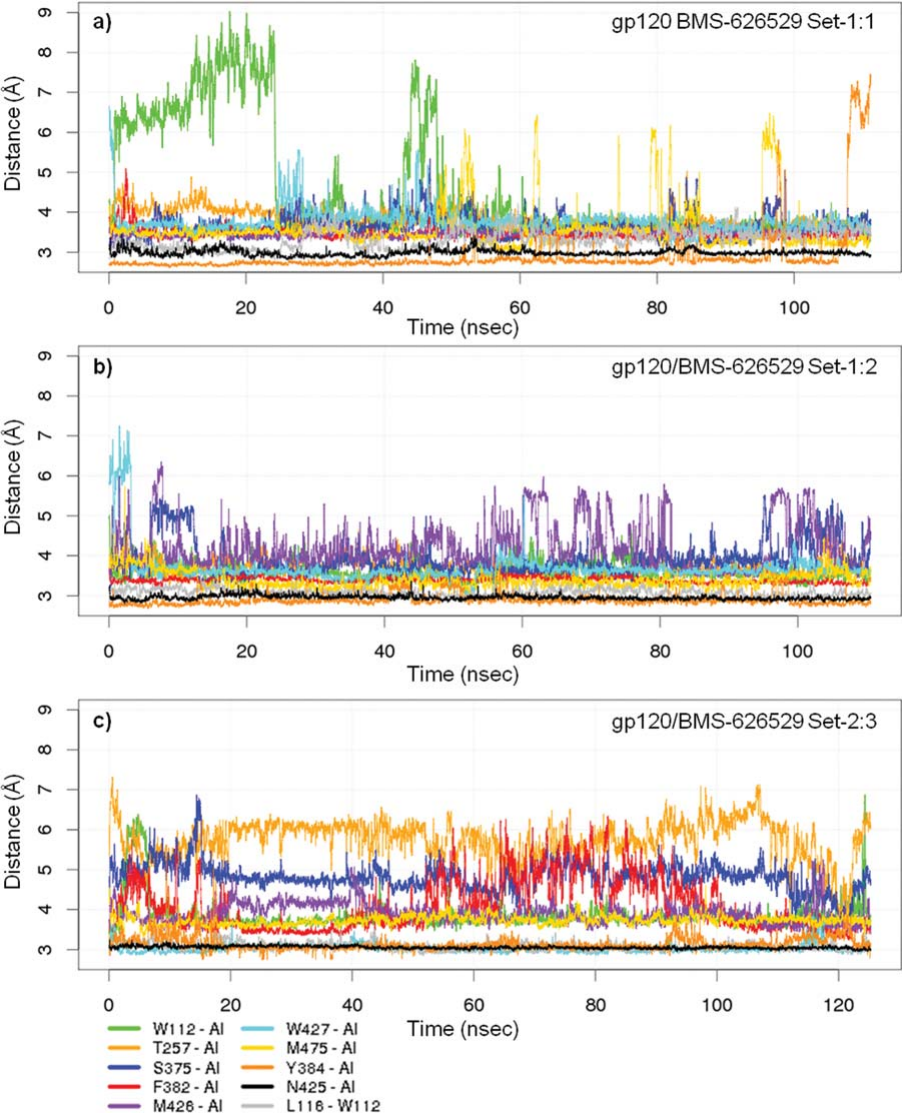
Distance plots for BMS-626529 and specific amino acids linked to key gp120 resistance substitutions. Molecular dynamics simulation a) gp120 UNLIG/BMS-626529 Set-1:1 binding pose, b) gp120 pCD4/BMS-626529 Set-1:2 binding pose, and c) gp120 pCD4/BMS-626529 Set-2:3 binding pose.

Distance plots for BMS-626529 and specific gp120 amino acids linked to key clinical and laboratory-generated resistance substitutions are shown in Figure 9. In the models, gp120 residues W112, T257, S375, F382, M426, W427, and M475 made direct contact with BMS-626529 throughout the simulations, while L116 made direct contact with W112, which in turn packed against the benzamide ring of BMS-626529. Mean distances between BMS-626529 and gp120 amino acids in the simulations are shown in Table I. The V68 and M434 residues lie outside of the binding site and distal to BMS-626529. While M434 is involved in bridging sheet packing (see below), V68 is on the outer edge of the inner domain and in close proximity to the region of gp41 that exits in the central cavity of the timer spike in the poorly formed CD4 binding site of the UNLIG state. Changes at V68 likely affect the projection and stability of the gp41 residues that spill into the CD4 binding site. Due to the very close proximity of gp41 and the β-turn residues of the β20–β21 sheet in the X-ray structure of the trimer spike, it is probable that gp41 influences the conformational sampling of the β20–β21 sheet and turn residues (Fig. 7).

**Table I:** Mean Distances Between BMS-626529 and gp120 Amino Acids During Molecular Dynamics Simulations Carried out Starting from Different Docked Poses of BMS-626529 Bound to the Unliganded (UNLIG) gp120 Model

	Set-1:1	Set-1:1	Set-1:2	Set-1:2	Set-2:3	Set-2:3	
Labels	Mean distance	SD	Mean distance	SD	Mean distance	SD	Description
**V68**	14.30	1.39	13.13	0.62	13.61	0.74	b
I108	5.26	1.17	4.54	0.61	4.30	0.41	
I109	6.28	1.45	4.07	0.47	3.46	0.21	
W112	4.55	1.51	3.59	0.34	3.85	0.49	c
D113	8.73	1.56	7.70	0.87	6.83	0.54	c
**L116**	9.01	1.32	8.90	1.38	8.03	0.60	b
A204	14.26	1.62	13.16	0.89	15.27	0.66	c
V255	3.34	0.31	3.51	0.28	3.97	0.39	
T257	3.84	0.33	3.68	0.30	5.73	0.59	c
D368	8.64	2.42	7.36	1.15	6.57	0.78	
P369	9.20	1.91	7.25	0.43	6.91	0.57	
E370	5.33	1.61	3.47	0.36	3.52	0.33	
I371	5.67	2.26	5.92	1.14	7.11	1.44	
**S375**	3.65	0.40	3.90	0.55	4.82	0.45	a,b
N377	6.36	0.40	6.36	0.55	7.21	0.86	c
F382	3.46	0.23	3.39	0.17	4.10	0.74	c
Y384	2.93	0.73	2.90	0.22	3.14	0.28	H-bond
K421	4.25	1.22	3.80	0.69	3.47	0.82	
I423	4.83	0.47	5.22	0.37	4.26	0.57	
I424	3.51	0.19	3.49	0.19	3.44	0.23	
N425	2.99	0.16	2.96	0.15	3.05	0.12	H-bond
**M426**	3.49	0.18	4.22	0.71	3.90	0.35	a,b
W427	3.77	0.38	3.68	0.47	3.03	0.20	c
**M434**	9.05	0.59	8.91	0.52	9.42	0.32	a
G473	3.47	0.62	3.64	0.48	4.03	0.76	
D474	4.73	0.94	3.79	0.70	5.19	1.06	
**M475**	3.59	0.62	3.38	0.30	3.73	0.26	a
I109—W427	4.35	1.68	3.85	0.37	3.72	0.21	d
W112—W427	6.35	2.91	7.78	0.84	7.72	0.71	d
V255—W427	7.91	0.74	7.24	1.11	7.06	0.59	d
M475—W427	4.13	0.66	3.85	0.78	3.64	0.27	d
L116—W112	3.34	0.32	3.08	0.18	3.08	0.19	

Contact distance is considered to be ≤5 Å.

^a^ = Site of clinically derived BMS-626529 resistance substitution.

^b^ = Site of clinically derived BMS-488043 resistance substitution.

^c^ = Site of laboratory-derived resistance substitution linked to reduced susceptibility to earlier AIs.

^d^ = W427 pocket.

The BMS-626529/gp120 interactions observed in the MD models are supported by previous site-directed mutagenesis studies. In the MD models, BMS-626529 extends over T257 and S375 and packs against the CD4-binding loop. In a study by Madani *et al*., substitutions at both T257 and S375 produced varying declines in viral susceptibility to an earlier AI, BMS-378806.^47^ The reduction in susceptibility was dependent on the substitution. For example, a >45-fold reduction in susceptibility (nondetectable inhibition) was observed when substitutions with large amino acids occurred at positions T257 or S375. Only 7-fold and 18-fold reductions in susceptibility occurred with smaller amino acids at positions T257A and T257G.^47^ These relative changes in susceptibility are consistent with the loss of a hydrogen bond between the CD4-binding loop and T257A (destabilizing the CD4-binding loop which packs the AI), and the additional loss of van der Waals contact between the AI and T257G. In addition, substitution with amino acids containing large side chains at either T257 or S375 would reduce the size and shape of the binding site in this model and would physically block AI binding.

In the MD models, W427 sits over the AI and π-stacks with the azaindole moiety periodically during the simulation. Site-specific substitutions at W427 are shown to either enhance the activity of AIs against HIV-1 (W427F; twofold)^47^ or lead to a complete loss in both sCD4 and AI binding (W427V).^14^ These changes would fit with a model in which the unliganded “closed” conformation of gp120 (UNLIG) is in equilibrium with pCD4, with residue W427 from the β20–β21 sheet moving in and out of the “liganded W427 pocket.”^46^ With a W427F substitution, the smaller phenylalanine residue would not completely fill the “liganded W427 pocket” and is predicted to shift the equilibrium toward the gp120 UNLIG state, which would favor AI binding as W427F could still π-stack with the azaindole moiety of the AI. Alternatively, substitution with a small, nonaromatic, and branched valine residue (W427V) is predicted to strongly shift the equilibrium away from the gp120 pCD4 and bCD4 states, thereby blocking CD4 binding. In this scenario, the smaller branched side chain would no longer make sufficient contact with the AI to support binding.

In line with the direct contact of residues W112 and F382 with BMS-626529 in the MD models, W112A and F382L substitutions are shown to cause a >45-fold decrease in susceptibility of HIV-1 to AIs (nondetectable inhibition). This decrease in susceptibility is consistent with a loss of interactions due to substitution with amino acids containing a small side chain.

Throughout the simulations, hydrogen bonds were intermittently formed between BMS-626529 and gp120 residues Y384 phenol and N425 backbone NH (Fig. 9, dark orange and black). While there is currently no experimental data to support the Y384 interactions, the N425A change resulted in a 3.7-fold improvement in susceptibility that may be explained by improved access to the backbone NH for hydrogen bonding, i.e. one of the N425 rotamers blocks the AI approach to the backbone NH.

In addition to the site-directed mutation data, data from a tryptophan fluorescence-quenching binding assay reported by Guo *et al*.^14^ showed a concentration-dependent reduction in gp120 tryptophan fluorescence upon titration of BMS-378806 into a solution containing monomeric gp120_JRFL_ protein. This leveled off at a ratio of approximately 1:1 BMS-378806 to monomeric gp120_JRFL_, further supporting a model in which BMS-626529 interacts with one or more tryptophan residues.

### MD models and clinically important substitutions

In an 8-day monotherapy study of BMS-626529 (administered as prodrug BMS-663068) in HIV-1 infected subjects, minimal change was seen in the susceptibility of the virus to BMS-626529. Also, during the course of the study there was no evidence of any known AI resistance mutations, as assessed by standard population-based phenotypic and genotypic approaches.^25^,^26^ Despite this, there were 6 of 48 subjects on study that were classified as nonresponders (defined as a viral load drop of <1 log_10_ copies/mL during treatment).^25^ Nonresponse was associated with lower baseline susceptibility to BMS-626529,^26^ and the key baseline substitutions in gp120 that contributed to this reduction in susceptibility were S375M, M426L, M434I and M475I.^27^ Of these, S375, M426, and M475 reside in the modeled binding site, while M434 is distal to the inhibitor. In the MD models, residues S375, M426, and M475 make direct contact with the AI throughout the MD simulation, with S375 lying under the C4-methoxy, M426 over the piperazine ring and M475 adjacent to the benzamide-piperazine moieties of BMS-626529. Changes at S375 should lead to a loss of van der Waals and potential hydrogen bonding interactions between BMS-626529 and gp120, and substitutions to amino acids with large side chains, for example, S375M, should physically hinder AI binding. Likewise, the change to amino acids with large branched side chains at positions 426 and 475, for example, M426L and M475I, would decrease the size of the binding site, which could reduce AI binding. The M434I substitution occurs outside of the AI binding site, but is present in the β21 strand of the β20–β21 sheet. In the UNLIG “closed state” the side chain of M434 packs against the side chains of V200, T202, I423 that are in the β3 and β20 strands, respectively, and forms two backbone hydrogen bonds with I201 and Q203 of β3 in forming the β3–β21 anti-parallel sheet. The M434 change to a branched side chain will minimally result in a distortion of the UNLIG “closed state” bridging sheet. This could shift the gp120 equilibrium away from an effective AI-binding conformation (gp120 UNLIG) and/or toward the pre-CD4-binding conformation (gp120 pCD4), thereby blocking formation of the AI binding site.

### Proposed model for binding of BMS-626529 to HIV-1 gp120 and proposed mechanism-of-action for BMS-626529

**Figure 10:**
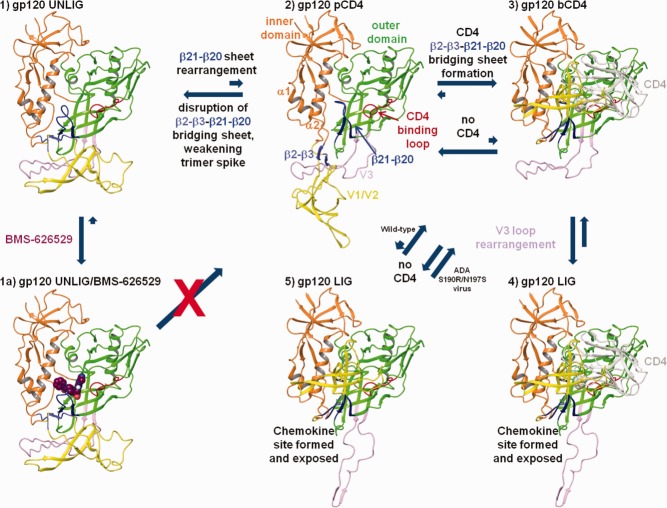
Proposed stepwise pathway for HIV-1 gp-120 attachment, CD4-independent attachment, and inhibition by attachment inhibitors. 1. In the gp120 UNLIG state the bridging sheet is ordered β2–β3-β21–β20 and directs the V1/V2 loops to pack against the V3 loop where they collectively form the quaternary structure of the trimer spike crown. In this state the β21–β20 sheet is misfolded for optimal CD4 binding. Note: the β21–β20 sheet is in equilibrium between nonCD4 and CD4 binding conformations with the nonCD4 binding conformations being the dominant form. 2. In the gp120 pre-CD4 state (pCD4) the β2–β3-β21–β20 bridging sheet is disrupted and the β21–β20 sheet is in equilibrium between nonCD4 and CD4 binding conformations. Based on SAXS data, this is likely to be the predominant form of the gp120 monomer in solution. Note that in the absence of CD4 (1), (2), (3), and (5) are in equilibrium but due to tertiary and quaternary structure involving the bridging sheet and the V1/V2/V3 loops (1) is far more prevalent; however, in the lab-adapted ADA S190R/N197S virus, the population of (2), (3) and (5) are elevated, leading to CD4-independent attachment via (5). 3. In the CD4-bound state (bCD4) CD4 stabilizes the β21–β20 sheet in the CD4 binding conformation and the re-ordered bridging sheet forms (β3–β2-β21–β20) moving the V1/V2 loops near CD4 domains I and II. 4. In the gp120 LIG state the V3 loop has opened to complete the formations and exposure of the chemokine binding site. 1a. BMS-626529 and related AIs are predicted to bind within the structurally conserved region in the outer domain of gp120 UNLIG (1) state. AIs binding within this pocket stabilize the UNLIG state by blocking the β21–β20 sheet from folding into a CD4-binding conformation and occupy the space next to the CD4 binding loop required for CD4:F43 binding, which blocks CD4 binding and the formation of the gp120 pCD4 state and all downstream events.

The transition between gp120 states in the homology models and the proposed mechanism of action of BMS-626529 are summarized in Figure 10. The initial unliganded conformation of gp120 (gp120 UNLIG, Fig. 10:1) is thought to be the predominant form in the gp120/gp41 trimer spike ensemble after cleavage of gp160 into gp120 and gp41.^7^,^11^,^32^,^35^–^37^,^75^ In the gp120 UNLIG state, the bridging sheet is ordered β2–β3-β21–β20, and the V1/V2 loop is packed against V3, forming the crown of the trimer spike. In this state, the β-turn between β20–β21 (residues V421, M426, W427) is near α2 (residues L116, W112, and D113) of the inner domain, respectively. This exposes a large hydrophobic cavity within the outer domain of gp120. In the UNLIG model, the CD4 binding site is only partially formed and CD4 is expected to bind weakly or not at all. Rearrangement of the β21–β20 sheet occurs during the transition between the gp120 UNLIG (Fig. 10:1) and gp120 pCD4 (Fig. 10:2) states. In the gp120 pCD4 state (Fig. 10:2) the β-turn residues of the β21–β20 sheet fill the hydrophobic pocket in the UNLIG outer domain and place W427 in the liganded W427 binding pocket. The conformational change in the β21–β20 sheet forms the gp120 water channel and CD4:F43 binding pocket, but at the cost of disrupting the unliganded bridging sheet. CD4 can readily be docked with the gp120 pCD4 state and would shift the equilibrium away from the UNLIG state toward the gp120 pCD4 state. The formation of the CD4/gp120 pCD4 complex results in stabilization of the CD4-binding loop and β20–β21 sheet.^7^,^12^,^13^ The stabilized disruption of the unliganded bridging sheet leads to opening of the V1/V2/V3 crown and the re-ordering of the bridging sheet to form the gp120/CD4 (bCD4, Fig. 10:3) complex. The formation of the gp120 bCD4 state (Fig. 10:3) marks the transition to the gp120 LIG state. In the gp120 bCD4/CD4 complex (Fig. 10:3), the β2–β3 sheet is zipped up against the stabilized β20–β21 sheet, forming the four-stranded (β3–β2-β21–β20) bridging sheet, and the V1/V2 loops are moved near CD4 domains 1 and 2. These changes result in the partial formation and exposure of the chemokine receptor-binding site. The final conformational changes occur during the formation of the gp120 LIG/CD4 complex (Fig. 10:4) and the V3 loop opens to expose fully the chemokine receptor-binding site. Based on these models, CD4 carries out three functions for viral entry: (1) CD4 binds with gp120 to attach the virus to the CD4+ T cell; (2) CD4 binding promotes the opening of the trimer complex by stabilizing the β20–β21 sheet in the “on” positions and the disrupted unliganded bridging sheet; and (3) CD4 provides a template to facilitate the formation of the re-ordered liganded bridging sheet. Due to the close proximity of the gp41 HR1 and FPPR regions to the CD4 binding site in the trimer complex, CD4 may also play a role in priming gp41 once the complex binds with the chemokine receptor.

Based on the homology models constructed, we have described a simple four-state pathway (UNLIG – pCD4 – bCD4 – LIG) that is supported by the recent smFRET results.^3^ However, it is clear from the number of antibody-bound states described in the literature^9^,^32^–^35^ and the absence of highly immunogenic structures, that the pathway between the unliganded (closed) and liganded (open) states is composed of a continuum of substates that are separated by a high transition barrier occurring between the UNLIG and LIG states. CD4 binding with gp120, or mutations in gp120 that lead to CD4-independent virus,^76^ lower the transition state barrier allowing gp120 to form the LIG conformation in which the co-receptor binding site is formed, exposed, and co-receptor binding can occur. In agreement with our model, the HDX data highlight the flexible nature of the unliganded states of gp120 (UNLIG and pCD4). Specifically, the V1/V2 stem (β2–β3), the β20–β21 sheet, CD4 binding loop, and the reduced flexibility in these regions following CD4 binding and formation of the gp120 bCD4 state.^7^,^12^,^13^

Following validation and refinement of BMS-626529-docked models, we propose that BMS-626529, and related AIs, bind within the structurally conserved hydrophobic cavity in the outer domain of gp120, present in the UNLIG (Fig. 10:1a) conformation of gp120, just underneath the β20–β21 sheet, and adjacent to the CD4 binding loop. In this binding model, the AI stabilizes the “closed” UNLIG state by preventing the closed-state bridging sheet from being disrupted. Although movement of the β20–β21 sheet β-turn residues (M426-V430, within the AI/gp120 UNLIG complex) can still occur, subsequent rearrangement of the β20–β21 sheet into the CD4-binding-competent gp120 pCD4 state is blocked. In addition, the overlay of CD4 onto the BMS-626529/gp120 UNLIG complex (Fig. 7) shows nearly complete overlap of the CD4:F43 side chain and the AI 7-(3-methyltriazole) azaindole moiety. There also is overlap when BMS-626529 is replaced with BMS-378806 (data not shown), but less so due to the smaller N7-azaindole. This model is consistent with direct inhibition of CD4 binding weakly to the nonoptimal CD4 binding site in the gp120 UNLIG state. The model also fits with previous data from Ho *et al*.,^15^ who suggested that binding of an AI can stabilize a conformation of gp120 that does not recognize CD4. Based on the homology modeling and compound docking reported in this study, it is likely that this conformation represents the gp120 UNLIG state.

A recent study by Li *et al*.^31^ demonstrated that laboratory-generated viruses that can infect cells in a CD4-independent manner are still susceptible to inhibition by BMS-626529. The proposed mechanism for CD4 independence in the ADA laboratory-generated virus^76^,^77^ suggests that the presence of the gp120 substitutions S190R and N197S shifts the equilibrium away from the native gp120 UNLIG (Fig. 10:1) state and toward the pCD4 (Fig. 10:2), bCD4 (Fig. 10:3), and gp120 LIG CD4-independent states (Fig. 10:5). It is postulated that this shift is due to the loss of the N197 glycosylation site and concomitant destabilization of the interactions between the V1/V2/V3 loops, which results in enhanced sampling of the V1/V2 loops. The increased sampling of the V1/V2 loops is likely to result in a large enough population of gp120 in the CD4-independent state (Fig. 10:5) where the stem of the V1/V2 loop (β2–β3 sheet) is complemented with the β20–β21 sheet in order to allow formation and unmasking of the chemokine receptor binding site and CD4-independent binding. Therefore, we propose that BMS-626529 can inhibit both CD4-induced and CD4-independent formation of the liganded “open state” four-stranded bridging sheet and the subsequent formation and exposure of the chemokine receptor binding site.

In conclusion, we have built a series of models based on known X-ray structures that represent different conformations of HIV-1 gp120. BMS-626529 was docked into the various models, which were refined and evaluated using MD simulations. The stable MD models, supported by AI SAR analyses, substitutions that affect susceptibility to AIs, and biochemical and biophysical data, were used to predict that BMS-626529 binds to the unliganded (UNLIG) gp120 structure within the structurally conserved outer domain, just underneath the β20–β21 sheet and adjacent to the CD4 binding loop. By binding to this site, we propose that BMS-626529 inhibits both CD4-induced and -independent formation of the four-stranded bridging sheet and subsequent formation and exposure of the co-receptor binding site. This unique mechanism of action prevents the initial interaction of the virus with CD4+ T cells and is distinct from that of currently available entry inhibitors, which target chemokine co-receptor binding and viral/cell membrane fusion, following binding of HIV-1 to the CD4 cell. This offers a novel therapeutic approach, broadening the range of antiretroviral therapies available for treatment of HIV-1 infection, particularly for patients with limited therapeutic options. BMS-626529 is currently being investigated clinically through the use of the prodrug BMS-663068, and a Phase IIb study of BMS-663068 in HIV-1-infected treatment-experienced subjects is ongoing (NCT01384734).
