# Supplementary material for: Homology models of the HIV-1 attachment inhibitor BMS-626529 bound to gp120 suggest a unique mechanism of action
Source: Proteins. 2014 Nov 17;83(2):331–50. doi: 10.1002/prot.24726 (PMC4681349; doi:10.1002/prot.24726)
Supplement: Supplementary file 1 — Supporting Information [file prot0083-0331-sd1.pdf]

Supporting Information

Figure S1. Mean structure from the Accelerated MD simulations on the Set-1 poses bound with gp120 UNLIG. Overlay of the mean structure. The structures are colored by MD thermal factors. The color scale runs from blue (most stable) to red (least stable). The inset shows that BMS-626529 has nearly converged between the four different simulations with the caveat that the proteins have not. The Set-1:1and 2 simulations show the tightest convergence. Set-1:3 and 4 differ somewhat from each other and from the Set-1:1 and 2 simulations.

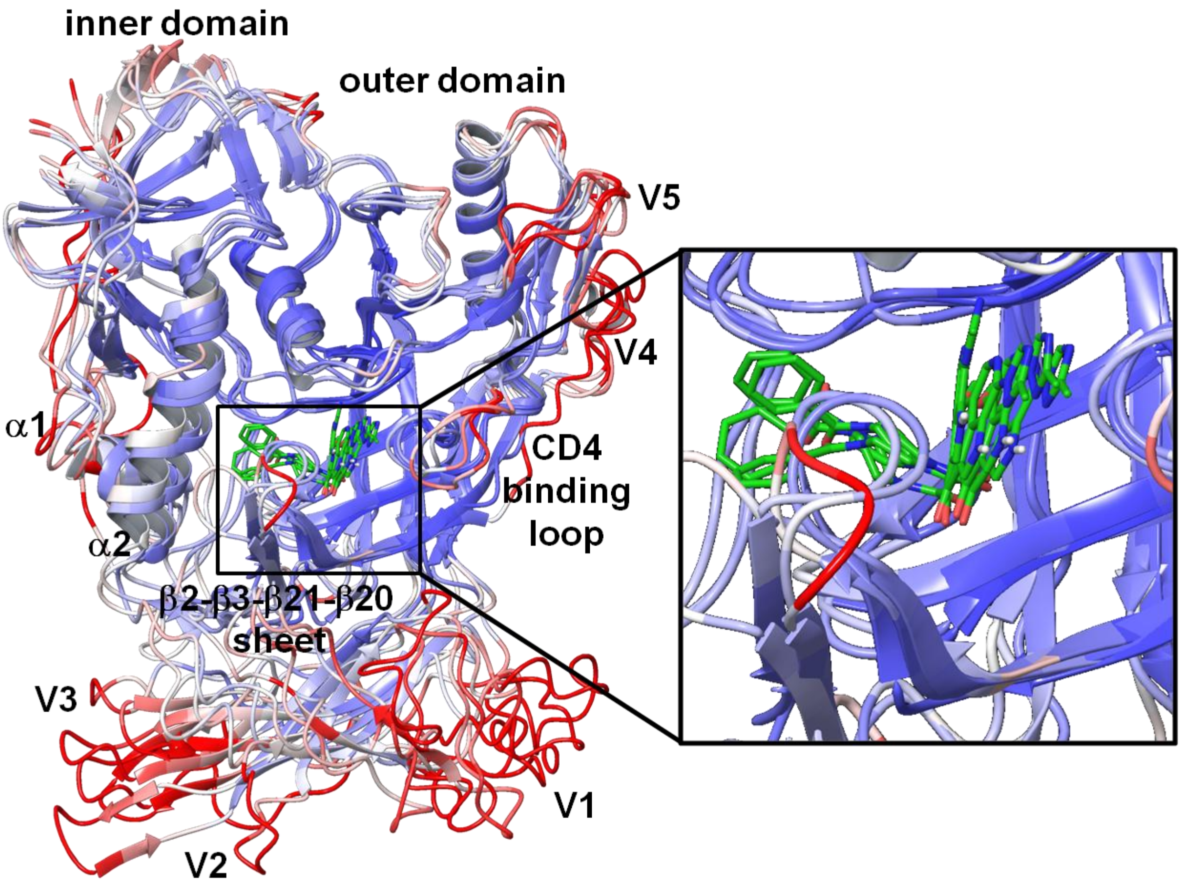

Figure S2: Mean structure from Set-1:1 and 2 simulations. Overlay of the mean structure from each simulation (averaged over 15–110-ns frames). The structures are colored by MD thermal factors. The color scale runs from blue (smallest fluctuation) to red (largest fluctuation). The inset shows that BMS-626529 has nearly converged between the two different simulations with the caveat that the proteins have not. Note that the piperazine ring is nearly flat in one structure and an up-chair in the other. The flat ring is due to averaging over multiple frames where nearly half the frames contain an up-chair while the other half contained the down-chair.

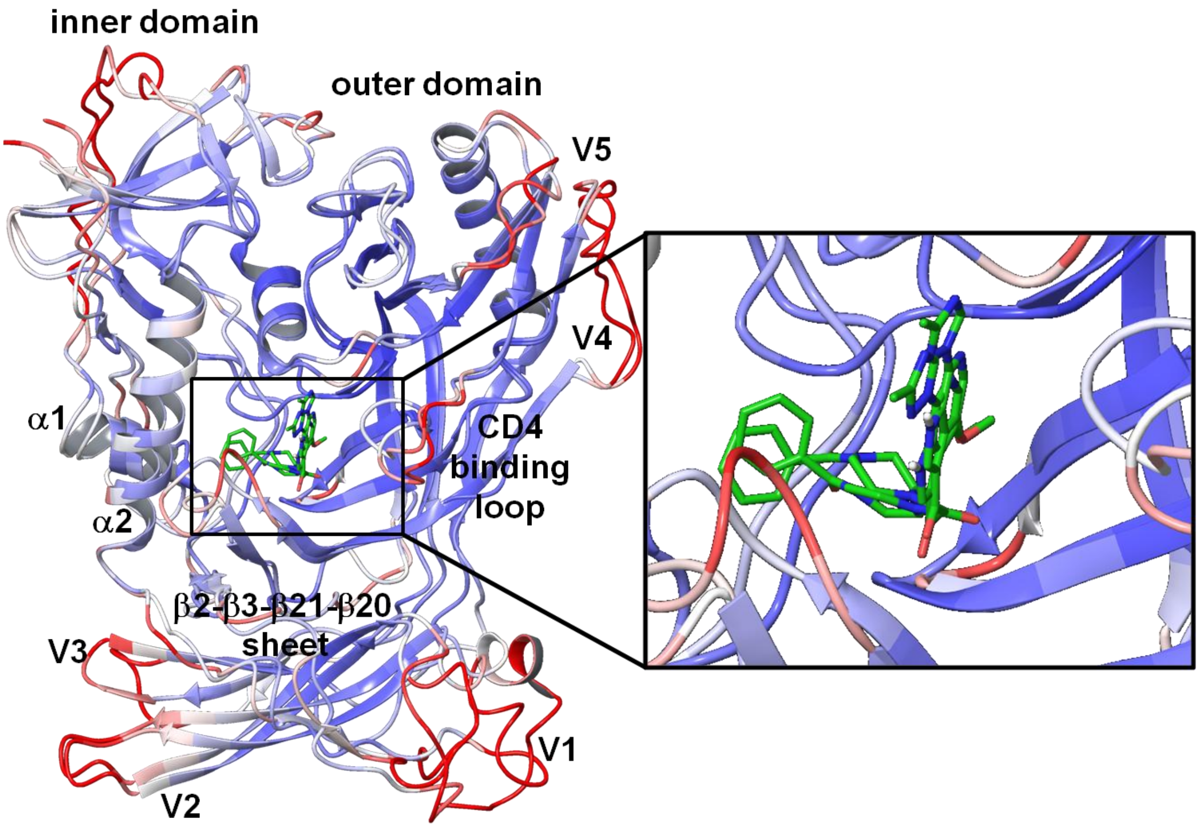

Videos S3, S4, and S5 portray the last 10 ns of the MD simulations of BMS-626529 bound to the gp120 “closed” UNLIG state, models Set-1:1, Set-1:2, and Set-2:3, respectively. PDB files were extracted in 500 ps intervals from the MD simulation and morphed from one another by creating three intermediate frames through linear interpolation using a Python script. The frames were rendered in PyMol (The PyMol Molecular Graphics System Version 1.5.0.4, Schrödinger LLC) and saved as PNG files. The PNG files were assembled into the video using MPlayer (MPlayer Version 1.1.0 MPlayer Team). Inner domain (orange), outer domain (green), V1/V2 (yellow), V3 (pink), β3–β2 sheet (light blue), β20–β21 (blue), CD4 binding loop (red), BMS-626529 (maroon). Yellow dotted lines depict hydrogen bonds.

Table S1: BMS-626529.pdb, BMS-626529.rtf, BMS-626529.prm files.

```
ATOM      1  C14 L0      1      -0.874  -1.932   8.836  1.00  0.00          L0
ATOM      2  H13 L0      1      -0.471  -1.880   7.828  1.00  0.00          L0
ATOM      3  C15 L0      1      -0.280  -1.194   9.855  1.00  0.00          L0
ATOM      4  H14 L0      1       0.610  -0.603   9.655  1.00  0.00          L0
ATOM      5  C16 L0      1      -0.833  -1.218  11.130  1.00  0.00          L0
ATOM      6  H15 L0      1      -0.352  -0.675  11.939  1.00  0.00          L0
ATOM      7  C17 L0      1      -2.004  -1.937  11.376  1.00  0.00          L0
ATOM      8  H16 L0      1      -2.472  -1.893  12.356  1.00  0.00          L0
ATOM      9  C18 L0      1      -2.574  -2.712  10.367  1.00  0.00          L0
ATOM     10  H17 L0      1      -3.472  -3.291  10.566  1.00  0.00          L0
ATOM     11  C19 L0      1      -1.988  -2.743   9.098  1.00  0.00          L0
ATOM     12  C13 L0      1      -2.544  -3.633   8.020  1.00  0.00          L0
ATOM     13  O3  L0      1      -3.731  -3.532   7.703  1.00  0.00          L0
ATOM     14  N2  L0      1      -1.674  -4.469   7.399  1.00  0.00          L0
ATOM     15  C12 L0      1      -0.365  -4.880   7.937  1.00  0.00          L0
ATOM     16  C10 L0      1       0.731  -4.366   6.979  1.00  0.00          L0
ATOM     17  H7  L0      1       1.708  -4.743   7.291  1.00  0.00          L0
ATOM     18  H8  L0      1       0.749  -3.273   6.982  1.00  0.00          L0
ATOM     19  H11 L0      1      -0.323  -5.970   8.006  1.00  0.00          L0
ATOM     20  H12 L0      1      -0.227  -4.450   8.932  1.00  0.00          L0
ATOM     21  C11 L0      1      -1.963  -5.048   6.085  1.00  0.00          L0
ATOM     22  H9  L0      1      -2.973  -4.776   5.769  1.00  0.00          L0
ATOM     23  H10 L0      1      -1.879  -6.137   6.127  1.00  0.00          L0
ATOM     24  C9  L0      1      -0.913  -4.458   5.120  1.00  0.00          L0
ATOM     25  H6  L0      1      -0.991  -3.368   5.098  1.00  0.00          L0
ATOM     26  H18 L0      1      -1.064  -4.851   4.111  1.00  0.00          L0
ATOM     27  N1  L0      1       0.426  -4.852   5.613  1.00  0.00          L0
ATOM     28  C8  L0      1       1.247  -5.580   4.803  1.00  0.00          L0
ATOM     29  O2  L0      1       0.944  -5.989   3.681  1.00  0.00          L0
ATOM     30  C7  L0      1       2.698  -5.909   5.345  1.00  0.00          L0
ATOM     31  O1  L0      1       3.024  -7.096   5.366  1.00  0.00          L0
ATOM     32  C1  L0      1       3.606  -4.868   5.848  1.00  0.00          L0
ATOM     33  C   L0      1       4.642  -5.169   6.712  1.00  0.00          L0
ATOM     34  H   L0      1       4.919  -6.123   7.144  1.00  0.00          L0
ATOM     35  N   L0      1       5.365  -4.056   7.010  1.00  0.00          L0
ATOM     36  H1  L0      1       6.163  -4.013   7.629  1.00  0.00          L0
ATOM     37  C3  L0      1       4.849  -2.974   6.339  1.00  0.00          L0
ATOM     38  C2  L0      1       3.709  -3.436   5.590  1.00  0.00          L0
ATOM     39  C5  L0      1       3.022  -2.465   4.818  1.00  0.00          L0
ATOM     40  O   L0      1       1.977  -2.899   4.034  1.00  0.00          L0
ATOM     41  C6  L0      1       0.961  -1.974   3.660  1.00  0.00          L0
ATOM     42  H3  L0      1       0.222  -2.492   3.044  1.00  0.00          L0
ATOM     43  H4  L0      1       1.409  -1.156   3.091  1.00  0.00          L0
ATOM     44  H5  L0      1       0.483  -1.579   4.560  1.00  0.00          L0
ATOM     45  C4  L0      1       3.483  -1.144   4.862  1.00  0.00          L0
ATOM     46  H2  L0      1       2.974  -0.366   4.297  1.00  0.00          L0
ATOM     47  N3  L0      1       4.553  -0.729   5.576  1.00  0.00          L0
ATOM     48  C20 L0      1       5.264  -1.632   6.296  1.00  0.00          L0
ATOM     49  N5  L0      1       6.436  -1.172   6.949  1.00  0.00          L0
ATOM     50  N4  L0      1       7.427  -1.996   7.320  1.00  0.00          L0
ATOM     51  C21 L0      1       8.327  -1.174   7.811  1.00  0.00          L0
ATOM     52  C23 L0      1       9.651  -1.681   8.331  1.00  0.00          L0
ATOM     53  H20 L0      1      10.127  -2.341   7.603  1.00  0.00          L0
ATOM     54  H21 L0      1       9.501  -2.243   9.255  1.00  0.00          L0
ATOM     55  H22 L0      1      10.324  -0.843   8.523  1.00  0.00          L0
ATOM     56  N6  L0      1       8.011   0.111   7.816  1.00  0.00          L0
ATOM     57  C22 L0      1       6.825   0.104   7.265  1.00  0.00          L0
ATOM     58  H19 L0      1       6.209   0.968   7.071  1.00  0.00          L0
END
```

```
* AMBER   ! Force Field for organic molecules
* Wang J, Wolf RM, Caldwell JW, Kollman PA, Case DA. Development and testing
* of a general amber force field. J Comput Chem 2004;25:1157-1174.
* Converted into CHARMM format
```

```
* David R. Langley, Feb. 3, 2006
*
   22    0

AUTOGENERATE ANGLES DIHE
DEFA FIRS NONE LAST NONE

RESI L0       0.00
GROUP
ATOM   C14    gca    -0.10772
ATOM   H13    gha    0.13831
ATOM   C15    gca    -0.13266
ATOM   H14    gha    0.13743
ATOM   C16    gca    -0.11489
ATOM   H15    gha    0.13587
ATOM   C17    gca    -0.13256
ATOM   H16    gha    0.13793
ATOM   C18    gca    -0.09041
ATOM   H17    gha    0.14654
ATOM   C19    gca    -0.12969
ATOM   C13    gc     0.67013
ATOM   O3     go     -0.59347
ATOM   N2     gn     -0.46277
ATOM   C12    gc3    0.06199
ATOM   C10    gc3    0.05916
ATOM   H7     gh1    0.08972
ATOM   H8     gh1    0.05167
ATOM   H11    gh1    0.06040
ATOM   H12    gh1    0.07036
ATOM   C11    gc3    0.05818
ATOM   H9     gh1    0.05548
ATOM   H10    gh1    0.09426
ATOM   C9     gc3    0.06105
ATOM   H6     gh1    0.06441
ATOM   H18    gh1    0.06219
ATOM   N1     gn     -0.44118
ATOM   C8     gc     0.59924
ATOM   O2     go     -0.57646
ATOM   C7     gc     0.55521
ATOM   O1     go     -0.51376
ATOM   C1     gcd    -0.24350
ATOM   C      gcc    -0.01573
ATOM   H      gh4    0.19607
ATOM   N      gna    -0.13879
ATOM   H1     ghn    0.33719
ATOM   C3     gca    -0.14933
ATOM   C2     gca    -0.03382
ATOM   C5     gca    0.03810
ATOM   O      gos    -0.31899
ATOM   C6     gc3    0.11206
ATOM   H3     gh1    0.08701
ATOM   H4     gh1    0.04505
ATOM   H5     gh1    0.02774
ATOM   C4     gca    0.33916
ATOM   H2     gh4    0.03706
ATOM   N3     gnb    -0.66193
ATOM   C20    gca    0.50358
ATOM   N5     gna    -0.09800
ATOM   N4     gnd    -0.56355
ATOM   C21    gcc    0.54846
ATOM   C23    gc3    -0.11818
ATOM   H20    ghc    0.07155
ATOM   H21    ghc    0.05781
ATOM   H22    ghc    0.05808
ATOM   N6     gnc    -0.68766
ATOM   C22    gcd    0.46264
ATOM   H19    gh5    0.09396
BOND   C      H
```

```
BOND   C      N
BOND   C      C1
BOND   N      H1
BOND   N      C3
BOND   C1     C2
BOND   C1     C7
BOND   C2     C3
BOND   C2     C5
BOND   C3     C20
BOND   C4     H2
BOND   C4     C5
BOND   C4     N3
BOND   C5     O
BOND   O      C6
BOND   C6     H3
BOND   C6     H4
BOND   C6     H5
BOND   C7     O1
BOND   C7     C8
BOND   C8     O2
BOND   C8     N1
BOND   N1     C9
BOND   N1     C10
BOND   C9     H6
BOND   C9     C11
BOND   C9     H18
BOND   C10    H7
BOND   C10    H8
BOND   C10    C12
BOND   C11    H9
BOND   C11    H10
BOND   C11    N2
BOND   C12    H11
BOND   C12    H12
BOND   C12    N2
BOND   N2     C13
BOND   C13    O3
BOND   C13    C19
BOND   C14    H13
BOND   C14    C15
BOND   C14    C19
BOND   C15    H14
BOND   C15    C16
BOND   C16    H15
BOND   C16    C17
BOND   C17    H16
BOND   C17    C18
BOND   C18    H17
BOND   C18    C19
BOND   N3     C20
BOND   C20    N5
BOND   C21    N4
BOND   C21    N6
BOND   C21    C23
BOND   N4     N5
BOND   C22    H19
BOND   C22    N5
BOND   C22    N6
BOND   C23    H20
BOND   C23    H21
BOND   C23    H22
IMPH   C19    C15    C14    H13
IMPH   C14    C16    C15    H14
IMPH   C15    C17    C16    H15
IMPH   C18    C16    C17    H16
IMPH   C19    C17    C18    H17
IMPH   C13    C18    C19    C14
IMPH   C19    N2     C13    O3
```

```
IMPH   C7     N1     C8     O2
IMPH   C8     C1     C7     O1
IMPH   C7     C2     C1     C
IMPH   C1     H      C      N
IMPH   C3     C      N      H1
IMPH   C2     C20    C3     N
IMPH   C3     C5     C2     C1
IMPH   C2     C4     C5     O
IMPH   C5     H2     C4     N3
IMPH   C3     N5     C20    N3
IMPH   C20    C22    N5     N4
IMPH   C23    N6     C21    N4
IMPH   H19    N5     C22    N6
IMPH    C8      C9      N1      C10 !DRL
IMPH    C13     C11     N2      C12 !DRL
END

* AMBER   ! Force Field for organic molecules
* Wang J, Wolf RM, Caldwell JW, Kollman PA, Case DA. Development and testing * of a
general amber force field. J Comput Chem 2004;25:1157-1174.
* Converted into CHARMM format
* David R. Langley, Feb. 3, 2006
*

BOND
 gc  gc    290.1   1.550
 gc  gn    478.2   1.345
 gc  go    648.0   1.214
 gc gc3    328.3   1.508
 gc gca    349.7   1.487
 gc gcc    377.4   1.462
 gc gcd    377.4   1.462
 gc gha    325.1   1.101
 gc gnc    438.8   1.371
 gc gnd    438.8   1.371
 gc gos    411.3   1.343
 gn  gn    469.7   1.390
 gn  go    646.6   1.264
 gn gna    486.8   1.379
 gn gnc    532.1   1.352
 gn gnd    532.1   1.352
 gn gos    372.3   1.429
 go  go    384.3   1.430
 go gos    306.3   1.504
gc3  gn    330.6   1.460
gc3  go    422.6   1.335
gc3 gc3    303.1   1.535
gc3 gca    323.5   1.513
gc3 gcc    337.3   1.499
gc3 gcd    337.3   1.499
gc3 gh1    335.9   1.093
gc3 ghc    337.3   1.092
gc3 gna    334.7   1.456
gc3 gnc    334.7   1.456
gc3 gnd    334.7   1.456
gc3 gos    301.5   1.439
gca  gn    372.3   1.422
gca  go    519.7   1.275
gca gca    478.4   1.387
gca gcc    411.7   1.434
gca gcd    411.7   1.434
gca gh4    342.9   1.088
gca gh5    347.2   1.085
gca gha    344.3   1.087
gca gna    470.3   1.350
gca gnb    483.1   1.342
gca gnc    492.9   1.336
gca gnd    492.9   1.336
```

```
gca gos    372.4   1.373
gcc  gn    426.0   1.380
gcc gcc    418.3   1.429
gcc gcd    504.0   1.371
gcc gh4    350.1   1.083
gcc gh5    356.0   1.079
gcc gha    347.2   1.085
gcc gna    438.8   1.371
gcc gnc    431.6   1.376
gcc gnd    494.6   1.335
gcc gos    376.1   1.370
gcd  gn    426.0   1.380
gcd gcd    418.3   1.429
gcd gh4    350.1   1.083
gcd gh5    356.0   1.079
gcd gha    347.2   1.085
gcd gna    438.8   1.371
gcd gnc    494.6   1.335
gcd gnd    431.6   1.376
gcd gos    376.1   1.370
ghn  gn    410.2   1.009
ghn gna    406.6   1.011
gna  go    644.3   1.265
gna gna    453.3   1.401
gna gnc    535.7   1.350
gna gnd    535.7   1.350
gna gos    355.2   1.444
gnb gnb    550.2   1.342
gnc gnc    486.8   1.379
gnc gnd    602.9   1.315
gnc gos    414.9   1.395
gnd gnd    486.8   1.379
gnd gos    414.9   1.395
gos gos    343.6   1.466

ANGLE
 gc  gc  gc   62.300 111.680
 gc  gc  gn   69.900 104.810
 gc  gc  go   66.800 122.340
 gc  gc gc3   61.700 116.860
 gc  gc gcc   64.000 111.670
 gc  gc gcd   64.000 111.670
 gc  gc gha   44.800 115.430
 gc  gc ghc   44.600 117.080
 gc  gn  gc   67.400 119.630
 gc  gn  gn   68.200 118.420
 gc  gn  go   71.600 118.900
 gc  gn gc3   63.900 121.350
 gc  gn gca   64.300 123.710
 gc  gn gcc   65.200 124.190
 gc  gn gcd   65.200 124.190
 gc  gn ghn   49.200 118.460
 gc  gn gna   68.200 119.200
 gc  gn gnc   68.600 120.310
 gc  gn gnd   68.600 120.310
 gc  gn gos   69.100 113.140
 gc gc3  gc   63.700 112.950
 gc gc3  gn   66.700 111.560
 gc gc3 gc3   63.800 110.530
 gc gc3 gca   64.100 111.230
 gc gc3 gh1   47.600 107.660
 gc gc3 ghc   47.200 109.680
 gc gc3 gna   68.600 105.720
 gc gc3 gos   68.000 109.820
 gc gca  gc   62.700 120.000
 gc gca gc3   62.600 118.060
 gc gca gca   64.600 120.140
 gc gca gha   46.500 115.900
```

gc	gca	ghc	11.200	120.000
gc	gca	gnc	64.400	130.800
gc	gca	gnd	64.400	130.800
gc	gcc	gc3	64.900	112.750
gc	gcc	gca	63.600	122.950
gc	gcc	gcc	63.700	122.690
gc	gcc	gcd	65.700	119.920
gc	gcc	gha	47.000	117.020
gc	gcc	gnc	64.300	130.800
gc	gcc	gnd	70.100	112.910
gc	gcd	gn	68.200	116.060
gc	gcd	gc3	64.900	112.750
gc	gcd	gca	63.600	122.950
gc	gcd	gcc	65.700	119.920
gc	gcd	gcd	63.700	122.690
gc	gcd	gha	47.000	117.020
gc	gcd	gnc	70.100	112.910
gc	gcd	gnd	64.300	130.800
gc	gna	gc	64.700	126.400
gc	gna	gn	66.300	124.660
gc	gna	gc3	64.700	117.600
gc	gna	gca	65.300	125.200
gc	gna	ghn	48.800	118.000
gc	gnc	gca	66.600	120.660
gc	gnc	gcd	67.000	119.250
gc	gnd	gca	66.600	120.660
gc	gnd	gcc	67.000	119.250
gc	gos	gc	64.000	122.650
gc	gos	gc3	63.600	115.140
gc	gos	gca	64.600	117.610
gc	gos	gcc	64.200	119.620
gc	gos	gcd	64.200	119.620
gn	gc	gn	75.400	111.700
gn	gc	go	75.800	122.030
gn	gc	gnc	73.000	116.840
gn	gc	gnd	73.000	116.840
gn	gc	gos	74.700	115.250
gn	gn	gn	69.900	114.620
gn	gc3	gn	68.800	113.810
gn	gca	gnc	69.700	123.860
gn	gca	gnd	69.700	123.860
gn	gcc	gc	68.200	116.060
gn	gcc	gnd	70.900	123.860
gn	gcd	gnc	70.900	123.860
gn	gna	gn	67.800	123.800
gn	gna	gnc	69.600	119.850
gn	gna	gnd	69.600	119.850
gn	gna	gos	72.300	104.710
gn	gos	gn	67.200	108.310
go	gc	go	79.100	127.330
go	gc	gos	76.200	122.430
go	gn	go	73.400	128.610
go	gc3	go	73.400	122.300
go	gca	go	75.500	126.820
go	gna	go	74.000	126.200
go	gna	gos	70.800	118.390
go	gos	go	62.800	114.680
gc3	gc	gn	67.900	115.150
gc3	gc	go	68.000	123.110
gc3	gc	gc3	62.800	116.050
gc3	gc	gca	62.300	119.530
gc3	gc	gcc	67.200	104.510
gc3	gc	gcd	67.200	104.510
gc3	gc	gha	46.000	115.220
gc3	gc	gos	69.300	111.960
gc3	gn	gc3	64.000	112.620
gc3	gn	gca	62.400	121.150
gc3	gn	gcc	63.300	121.170

gc3	gn	gcd	63.300	121.170
gc3	gn	ghn	46.000	116.780
gc3	gc3	gn	65.900	112.130
gc3	gc3	gc3	63.200	110.630
gc3	gc3	gca	62.500	114.610
gc3	gc3	gcc	64.700	108.100
gc3	gc3	gcd	64.700	108.100
gc3	gc3	gh1	46.400	110.070
gc3	gc3	ghc	46.400	110.050
gc3	gc3	gna	65.800	112.590
gc3	gc3	gos	67.800	108.420
gc3	gca	gc3	62.400	116.800
gc3	gca	gca	63.800	120.630
gc3	gcc	gca	64.100	117.810
gc3	gcc	gcc	64.700	115.970
gc3	gcc	gcd	64.800	119.450
gc3	gcc	gha	45.100	121.520
gc3	gcc	gna	65.500	122.800
gc3	gcc	gnc	65.800	121.440
gc3	gcc	gnd	66.400	121.960
gc3	gcc	gos	67.800	115.890
gc3	gcd	gca	64.100	117.810
gc3	gcd	gcc	64.800	119.450
gc3	gcd	gcd	64.700	115.970
gc3	gcd	gha	45.100	121.520
gc3	gcd	gna	65.500	122.800
gc3	gcd	gnc	66.400	121.960
gc3	gcd	gnd	65.800	121.440
gc3	gcd	gos	67.800	115.890
gc3	gna	gc3	60.700	125.590
gc3	gna	gca	63.100	124.360
gc3	gna	gcc	62.600	125.090
gc3	gna	gcd	62.600	125.090
gc3	gna	gnc	65.700	120.460
gc3	gna	gnd	65.700	120.460
gc3	gna	gos	68.900	104.390
gc3	gnc	gcd	67.600	109.510
gc3	gnd	gcc	67.600	109.510
gc3	gos	gn	65.300	108.570
gc3	gos	go	65.600	103.000
gc3	gos	gc3	62.100	113.410
gc3	gos	gca	62.400	117.600
gc3	gos	gcc	63.100	115.180
gc3	gos	gcd	63.100	115.180
gc3	gos	gna	64.500	109.910
gc3	gos	gnc	64.800	112.730
gc3	gos	gnd	64.800	112.730
gc3	gos	gos	65.900	105.010
gca	gc	gn	69.400	112.030
gca	gc	go	68.700	123.440
gca	gc	gca	62.800	119.530
gca	gc	gcc	64.800	114.090
gca	gc	gcd	64.800	114.090
gca	gc	gha	46.800	114.120
gca	gc	gos	68.800	115.540
gca	gn	gca	64.500	116.800
gca	gn	gcc	65.000	118.420
gca	gn	gcd	65.000	118.420
gca	gn	ghn	47.600	114.590
gca	gn	gna	66.800	117.570
gca	gn	gnc	68.800	113.030
gca	gn	gnd	68.800	113.030
gca	gc3	gca	63.600	112.470
gca	gc3	gcc	65.200	108.080
gca	gc3	gcd	65.200	108.080
gca	gc3	gh1	46.800	110.950
gca	gc3	ghc	47.000	110.150
gca	gc3	gnc	68.200	106.510

```
gca gc3 gnd   68.200 106.510
gca gc3 gos   67.700 110.510
gca gca  gn   68.000 119.890
gca gca  go   70.900 123.430
gca gca gca   67.200 119.970
gca gca gcc   66.000 120.100
gca gca gcd   66.000 120.100
gca gca gh4   48.200 121.090
gca gca gha   48.500 120.010
gca gca gna   70.200 118.340
gca gca gnb   69.200 122.630
gca gca gnc   70.100 119.720
gca gca gnd   70.100 119.720
gca gca gos   69.800 119.200
gca gcc  gn   72.500 104.870
gca gcc gcc   67.700 111.040
gca gcc gcd   68.200 113.510
gca gcc gh4   45.600 128.660
gca gcc gha   46.400 124.040
gca gcc gnd   70.200 115.050
gca gcc gos   69.500 116.240
gca gcd  gn   72.500 104.870
gca gcd gcc   68.200 113.510
gca gcd gcd   67.700 111.040
gca gcd gh4   45.600 128.660
gca gcd gha   46.400 124.040
gca gcd gnc   70.200 115.050
gca gcd gos   69.500 116.240
gca gna  gn   67.300 122.000
gca gna  go   70.900 120.680
gca gna gca   67.100 119.800
gca gna gcc   68.500 113.150
gca gna gcd   68.500 113.150
gca gna ghn   48.200 122.770
gca gna gna   66.300 123.760
gca gna gnc   69.300 117.850
gca gna gnd   69.300 117.850
gca gna gos   69.700 109.460
gca gnb gca   68.600 115.860
gca gnb gnb   69.400 118.890
gca gnc  gn   73.800 104.690
gca gnc gca   70.700 109.950
gca gnc gcd   71.100 108.820
gca gnc gna   74.100 104.160
gca gnc gos   73.100 104.340
gca gnd  gn   73.800 104.690
gca gnd gca   70.700 109.950
gca gnd gcc   71.100 108.820
gca gnd gna   74.100 104.160
gca gnd gos   73.100 104.340
gca gos gca   63.600 118.960
gca gos gcc   66.200 109.770
gca gos gcd   66.200 109.770
gca gos gna   66.500 108.240
gca gos gnc   66.100 113.680
gca gos gnd   66.100 113.680
gcc  gc  gn   70.200 111.860
gcc  gc  go   68.900 125.710
gcc  gc gcc   65.300 114.450
gcc  gc gcd   65.700 112.790
gcc  gc gos   70.500 112.300
gcc  gn  gn   66.500 121.370
gcc  gn  go   70.100 120.540
gcc  gn gcc   68.800 108.920
gcc  gn ghn   47.500 121.520
gcc  gn gna   67.900 117.570
gcc  gn gnc   69.900 113.030
gcc  gn gos   67.600 115.560
```

```
gcc gc3 gcc   65.500 107.990
gcc gc3 gcd   67.300 102.350
gcc gc3 gh1   47.000 111.620
gcc gc3 ghc   47.200 110.860
gcc gc3 gnc   68.400 107.040
gcc gc3 gos   67.800 111.280
gcc gca gnb   66.900 126.210
gcc gcc  gn   67.900 119.890
gcc gcc gcc   67.900 110.700
gcc gcc gcd   68.200 114.190
gcc gcc gh4   45.600 129.470
gcc gcc gha   46.600 123.740
gcc gcc gna   72.200 106.800
gcc gcc gnc   70.000 113.420
gcc gcc gnd   71.100 112.560
gcc gcc gos   68.400 120.430
gcc gcd  gn   70.700 115.520
gcc gcd gcc   69.300 115.510
gcc gcd gcd   68.200 114.190
gcc gcd gh4   47.200 129.110
gcc gcd gha   48.400 122.890
gcc gcd gna   72.900 109.420
gcc gcd gnd   71.000 114.980
gcc gcd gos   70.000 120.300
gcc gna  gn   66.500 123.190
gcc gna  go   69.000 125.210
gcc gna gcc   68.900 109.900
gcc gna gcd   63.900 128.010
gcc gna ghn   47.200 124.660
gcc gna gna   65.900 123.430
gcc gna gnc   70.200 113.020
gcc gna gos   67.300 115.740
gcc gnc gcc   68.600 110.190
gcc gnc gcd   70.500 107.470
gcc gnc gna   73.400 102.970
gcc gnc gnd   72.500 107.940
gcc gnd  gn   74.000 104.150
gcc gnd gcd   70.500 107.470
gcc gnd gna   74.200 103.730
gcc gnd gnd   71.400 109.510
gcc gnd gos   72.200 106.990
gcc gos gcc   66.500 109.170
gcc gos gcd   62.700 122.990
gcc gos gna   65.500 111.660
gcc gos gnc   68.100 107.230
gcc gos gos   66.300 108.470
gcd  gc  gn   70.200 111.860
gcd  gc  go   68.900 125.710
gcd  gc gcd   65.300 114.450
gcd  gc gos   70.500 112.300
gcd  gn  gn   66.500 121.370
gcd  gn  go   70.100 120.540
gcd  gn gcd   68.800 108.920
gcd  gn ghn   47.500 121.520
gcd  gn gna   67.900 117.570
gcd  gn gnd   69.900 113.030
gcd  gn gos   67.600 115.560
gcd gc3 gcd   65.500 107.990
gcd gc3 gh1   47.000 111.620
gcd gc3 ghc   47.200 110.860
gcd gc3 gnd   68.400 107.040
gcd gc3 gos   67.800 111.280
gcd gca gnb   66.900 126.210
gcd gcc  gn   70.700 115.520
gcd gcc gcd   69.300 115.510
gcd gcc gh4   47.200 129.110
gcd gcc gha   48.400 122.890
gcd gcc gna   72.900 109.420
```

gcd	gcc	gnc	71.000	114.980
gcd	gcc	gos	70.000	120.300
gcd	gcd	gn	67.900	119.890
gcd	gcd	gcd	67.900	110.700
gcd	gcd	gh4	45.600	129.470
gcd	gcd	gha	46.600	123.740
gcd	gcd	gna	72.200	106.800
gcd	gcd	gnc	71.100	112.560
gcd	gcd	gnd	70.000	113.420
gcd	gcd	gos	68.400	120.430
gcd	gna	gn	66.500	123.190
gcd	gna	go	69.000	125.210
gcd	gna	gcd	68.900	109.900
gcd	gna	ghn	47.200	124.660
gcd	gna	gna	65.900	123.430
gcd	gna	gnd	70.200	113.020
gcd	gna	gos	67.300	115.740
gcd	gnc	gn	74.000	104.150
gcd	gnc	gna	74.200	103.730
gcd	gnc	gnc	71.400	109.510
gcd	gnc	gos	72.200	106.990
gcd	gnd	gcd	68.600	110.190
gcd	gnd	gna	73.400	102.970
gcd	gnd	gnc	72.500	107.940
gcd	gos	gcd	66.500	109.170
gcd	gos	gna	65.500	111.660
gcd	gos	gnd	68.100	107.230
gcd	gos	gos	66.300	108.470
gh1	gc3	gn	49.800	109.320
gh1	gc3	go	52.000	117.190
gh1	gc3	gh1	39.200	109.550
gh1	gc3	gna	49.900	109.450
gh1	gc3	gnc	50.100	108.570
gh1	gc3	gnd	50.100	108.570
gh1	gc3	gos	50.800	108.820
gh4	gca	gn	49.500	116.020
gh4	gca	gna	51.900	114.650
gh4	gca	gnb	51.800	115.940
gh4	gca	gnc	51.500	118.360
gh4	gca	gnd	51.500	118.360
gh4	gca	gos	52.300	111.150
gh4	gcc	gn	50.400	117.620
gh4	gcc	gna	50.200	119.660
gh4	gcc	gnc	50.000	120.030
gh4	gcc	gnd	51.400	119.110
gh4	gcc	gos	52.300	111.890
gh4	gcd	gn	50.400	117.620
gh4	gcd	gna	50.200	119.660
gh4	gcd	gnc	51.400	119.110
gh4	gcd	gnd	50.000	120.030
gh4	gcd	gos	52.300	111.890
gh5	gca	gnb	51.800	116.350
gh5	gca	gnc	50.700	122.110
gh5	gca	gnd	50.700	122.110
gh5	gcc	gna	49.800	122.100
gh5	gcc	gnc	49.300	123.700
gh5	gcc	gnd	50.100	125.380
gh5	gcc	gos	51.300	116.330
gh5	gcd	gna	49.800	122.100
gh5	gcd	gnc	50.100	125.380
gh5	gcd	gnd	49.300	123.700
gh5	gcd	gos	51.300	116.330
gha	gc	gn	52.400	112.370
gha	gc	go	54.300	121.940
gha	gc	gha	37.900	115.610
gha	gc	gos	53.200	110.340
gha	gcc	gna	49.800	121.500
gha	gcc	gnc	50.700	116.540

gha	gcc	gnd	51.400	118.880
gha	gcc	gos	52.500	110.860
gha	gcd	gna	49.800	121.500
gha	gcd	gnc	51.400	118.880
gha	gcd	gnd	50.700	116.540
gha	gcd	gos	52.500	110.860
ghc	gc	gn	51.100	120.000
ghc	gc	go	55.400	120.000
ghc	gc	ghc	39.300	115.680
ghc	gc	gos	52.900	113.580
ghc	gc3	gn	49.800	109.500
ghc	gc3	ghc	39.400	108.350
ghc	gc3	gna	49.900	109.500
ghc	gc3	gos	50.900	108.700
ghn	gn	gn	50.100	113.120
ghn	gn	go	53.800	116.320
ghn	gn	ghn	39.700	117.850
ghn	gn	gna	50.400	113.550
ghn	gn	gos	50.100	108.900
ghn	gna	ghn	39.800	116.800
ghn	gna	gnc	50.000	119.610
ghn	gna	gnd	50.000	119.610
ghn	gna	gos	51.400	101.410
gna	gc	go	75.000	122.850
gna	gc	gna	73.200	115.400
gna	gn	gna	69.600	117.380
gna	gc3	gna	69.100	113.490
gna	gc3	gos	71.200	109.190
gna	gca	gna	74.500	113.320
gna	gca	gnb	70.200	128.560
gna	gcc	gnc	69.700	125.320
gna	gcc	gnd	74.800	112.020
gna	gcd	gnc	74.800	112.020
gna	gcd	gnd	69.700	125.320
gna	gna	gna	66.800	123.600
gna	gna	gnc	69.100	119.640
gna	gna	gnd	69.100	119.640
gna	gna	gos	70.200	109.470
gna	gnc	gnd	76.000	105.470
gna	gnd	gnc	76.000	105.470
gna	gos	gna	66.100	109.590
gnb	gca	gnb	71.200	125.840
gnb	gca	gnc	71.100	126.500
gnb	gca	gnd	71.100	126.500
gnb	gnb	gnb	70.400	121.040
gnc	gc	go	73.900	125.290
gnc	gn	gnc	71.300	116.410
gnc	gc3	gnc	70.000	110.610
gnc	gc3	gos	69.300	115.410
gnc	gca	gnc	70.700	128.740
gnc	gcc	gnd	72.500	118.690
gnc	gcd	gnd	72.500	118.690
gnc	gcd	gos	73.000	119.140
gnc	gna	go	72.000	122.790
gnc	gna	gnc	71.200	117.080
gnc	gna	gos	68.300	119.650
gnc	gnc	gnd	73.200	111.150
gnc	gnd	gnd	73.200	111.150
gnc	gnd	gos	74.400	107.220
gnc	gos	gnc	68.200	110.400
gnd	gc	go	73.900	125.290
gnd	gn	gnd	71.300	116.410
gnd	gc3	gnd	70.000	110.610
gnd	gc3	gos	69.300	115.410
gnd	gca	gnd	70.700	128.740
gnd	gcc	gos	73.000	119.140
gnd	gna	go	72.000	122.790
gnd	gna	gnd	71.200	117.080

```
gnd gna gos   68.300 119.650
gnd gnc gos   74.400 107.220
gnd gos gnd   68.200 110.400
gos  gc gos   77.800 107.490
gos  gn gos   71.300 106.530
gos gc3 gos   71.700 110.240
gos gca gos   74.000 113.730
gos gna gos   71.300 104.450

DIHE
  X   gc  gc   X  0.3000000000 2   180.0
  X   gc  gn   X  2.5000000000 2   180.0
  X   gc gc3   X  0.0000000000 2   180.0
  X   gc gca   X  3.6250000000 2   180.0
  gn  gc gca gca  0.6250000000 2   180.0 !DRL
  go  gc gca gca  0.6250000000 2   180.0 !DRL
  X   gc gcc   X  2.8750000000 2   180.0
  X   gc gcd   X  2.8750000000 2   180.0
  X   gc gna   X  0.3500000000 4   180.0
  X   gc gna   X  1.4500000000 2   180.0
  X   gc gnc   X  4.0000000000 2   180.0
  X   gc gnd   X  4.0000000000 2   180.0
  X   gc gos   X  2.7000000000 2   180.0
  X   gn  gn   X  1.1500000000 2     0.0
  X   gn gcc   X  1.6500000000 2   180.0
  X   gn gcd   X  1.6500000000 2   180.0
  X   gn gna   X  0.7000000000 2     0.0
  X   gn gnc   X  4.8000000000 2   180.0
  X   gn gnd   X  4.8000000000 2   180.0
  X   gn gos   X  1.1000000000 2     0.0
  X  gc3  gn   X  0.0000000000 2     0.0
  X  gc3 gc3   X  0.1555555556 3     0.0
  X  gc3 gca   X  0.0000000000 2     0.0
  X  gc3 gcc   X  0.0000000000 3     0.0
  X  gc3 gcd   X  0.0000000000 3     0.0
  X  gc3 gna   X  0.0000000000 2     0.0
  X  gc3 gos   X  0.3833333333 3     0.0
  X  gca  gn   X  0.4500000000 2   180.0
  X  gca gca   X  3.6250000000 2   180.0
  X  gca gna   X  1.1000000000 2   180.0 !DRL
  X  gca gnb   X  4.8000000000 2   180.0
  X  gca gnc   X  4.8000000000 2   180.0
  X  gca gnd   X  4.8000000000 2   180.0
  X  gca gos   X  0.9000000000 2   180.0
  X  gcc gcc   X  4.0000000000 2   180.0
  X  gcc gcd   X  4.0000000000 2   180.0
  X  gcc gna   X  1.7000000000 2   180.0
  X  gcc gnc   X  4.7500000000 2   180.0
  X  gcc gnd   X  4.7500000000 2   180.0
  X  gcd gcd   X  4.0000000000 2   180.0
  X  gcd gna   X  1.7000000000 2   180.0
  X  gcd gnc   X  4.7500000000 2   180.0
  X  gcd gnd   X  4.7500000000 2   180.0
  X  gna gna   X  0.9000000000 2     0.0
  X  gna gnc   X  4.8000000000 2   180.0
  X  gna gnd   X  4.8000000000 2   180.0
  X  gna gos   X  0.6500000000 2     0.0
  X  gnc gnc   X  4.0000000000 2   180.0
  X  gnc gnd   X  4.0000000000 2   180.0
  X  gnc gos   X  4.8000000000 2   180.0
  X  gnd gnd   X  4.0000000000 2   180.0
  X  gos gos   X  1.0000000000 1     0.0
 gc  gn gc3  gc 0.8000000000 1     0.0
 gc  gn gc3  gc 0.8500000000 2   180.0
 gc gcd gca gca 2.5500000000 2   180.0
 gn gc3  gc  gn 1.7000000000 1   180.0
 gn gc3  gc  gn 2.0000000000 2   180.0
 go  gc gos gc3 1.4000000000 1   180.0
```

```
 go  gc gos gc3 2.7000000000 2   180.0
gc3 gc3  gc  gn 0.0700000000 2     0.0
gc3 gc3  gc  gn 0.1000000000 4     0.0
gc3 gc3  gn  gc 0.1500000000 3   180.0
gc3 gc3  gn  gc 0.5000000000 4   180.0
gc3 gc3  gn  gc 0.5300000000 1     0.0
gc3 gc3 gc3 gc3 0.1800000000 3     0.0
gc3 gc3 gc3 gc3 0.2000000000 1   180.0
gc3 gc3 gc3 gc3 0.2500000000 2   180.0
gc3 gc3 gos  gc 0.3830000000 3     0.0
gc3 gc3 gos  gc 0.8000000000 1   180.0
gc3 gc3 gos gc3 0.1000000000 2   180.0
gc3 gc3 gos gc3 0.3830000000 3     0.0
gc3 gos gc3 gna 0.3830000000 3     0.0
gc3 gos gc3 gna 0.6500000000 2     0.0
gc3 gos gc3 gos 0.1000000000 3     0.0
gc3 gos gc3 gos 0.8500000000 2   180.0
gc3 gos gc3 gos 1.3500000000 1   180.0
gca gca gna gnd 1.5000000000 2   180.0 !DRL
gcc gcd gca gca 2.5500000000 2   180.0
gh1 gc3  gc  go 0.0800000000 3   180.0
gh1 gc3  gc  go 0.8000000000 1     0.0
gh1 gc3 gc3 gos 0.2500000000 1     0.0
ghc gc3  gc  go 0.0800000000 3   180.0
ghc gc3  gc  go 0.8000000000 1     0.0
ghc gc3 gc3 gc3 0.1600000000 3     0.0
ghc gc3 gc3 ghc 0.1500000000 3     0.0
ghc gc3 gc3 gos 0.2500000000 1     0.0
ghn  gn  gc  go 2.0000000000 1     0.0
ghn  gn  gc  go 2.5000000000 2   180.0
gnb gca gna gnd 1.5000000000 2   180.0 !DRL
gos gc3 gc3 gos 0.1440000000 3     0.0
gos gc3 gc3 gos 1.1750000000 2     0.0
gc3  gn gc3 gc3 0.7          3     0.0 !DRL

IMPR
  X   X  gc  go     10.500   2   180
  X   X  gn ghn      1.100   2   180
  X   X gca gha      1.100   2   180
  X   X gca ghc      1.100   2   180
  X   X gna ghn      1.100   2   180
  X   X  gn gc3     10.500   2   180
  X  go  gc  go     10.500   2   180
  X gc3  gn gc3     10.500   2   180
 gc gc3  gn  go      1.100   2   180
 gc gc3  gn ghn      1.100   2   180
 gc gca gca gc3      1.100   2   180
 gc gca gca gca      1.100   2   180
 gc gca gcd gcc      1.100   2   180
X X gc gos 1.100 2 180
X X gca gca 1.100 2 180
X X gca gcc 1.100 2 180
X X gca gcd 1.100 2 180
X X gca gn 1.100 2 180
X X gca gna 1.100 2 180
X X gca gnb 1.100 2 180
X X gca gnc 1.100 2 180
X X gca gnd 1.100 2 180
X X gca gos 1.100 2 180
X X gcc gcd 1.100 2 180
X X gcc gha 1.100 2 180
X X gcc gn 1.100 2 180
X X gcc gna 1.100 2 180
X X gcc gnc 1.100 2 180
X X gcc gnd 1.100 2 180
X X gcc go 1.100 2 180
X X gcc gos 1.100 2 180
X X gcd gcc 1.100 2 180
```

```
X X gcd gcd 1.100 2 180
X X gcd gha 1.100 2 180
X X gcd gn 1.100 2 180
X X gcd gna 1.100 2 180
X X gcd gnc 1.100 2 180
X X gcd gnd 1.100 2 180
X X gcd go 1.100 2 180
X X gcd gos 1.100 2 180
X X gn gc 1.100 2 180
X X gn gca 1.100 2 180
X X gn gcc 1.100 2 180
X X gn gcd 1.100 2 180
X X gn gn 1.100 2 180
X X gna gca 1.100 2 180
X X gna gcc 1.100 2 180
X X gna gcd 1.100 2 180
X X gna gnc 1.100 2 180
X X gna gnd 1.100 2 180
gc3 gca gca gna      1.100   2   180
gc3 gnc gcc gnd      1.100   2   180
gca  gc gca gc3      1.100   2   180
gca gca gca gc3      1.100   2   180
gca gca gca gcd      1.100   2   180
gca gca gca gna      1.100   2   180
gca gca gca gos      1.100   2   180
gca gca gna gc3      1.100   2   180
gca gcd gna gnd      1.100   2   180
gca gh4 gca gnb      1.100   2   180
gca ghc  gc  go      1.100   2   180
gca gna gca gnb      1.100   2   180
gcd gh4 gcc gna      1.100   2   180
gh5 gna gcd gnc      1.100   2   180
ghc  go  gc gos      1.100   2   180

 NONBONDED  NBXMOD 5  FSHIFT VSWITCH CDIEL -
     CUTNB 14.0  CTOFNB 12.0  CTONNB 10.0  EPS 1.0  E14FAC 0.83333333  WMIN 1.4

 gc   0  -0.08600  1.90800   0 -0.04300  1.90800
 gn   0  -0.17000  1.82400   0 -0.08500  1.82400
 go   0  -0.21000  1.66120   0 -0.10500  1.66120
gc3   0  -0.10940  1.90800   0 -0.05470  1.90800
gca   0  -0.08600  1.90800   0 -0.04300  1.90800
gcc   0  -0.08600  1.90800   0 -0.04300  1.90800
gcd   0  -0.08600  1.90800   0 -0.04300  1.90800
gh1   0  -0.01570  1.38700   0 -0.00785  1.38700
gh4   0  -0.01500  1.40900   0 -0.00750  1.40900
gh5   0  -0.01500  1.35900   0 -0.00750  1.35900
gha   0  -0.01500  1.45900   0 -0.00750  1.45900
ghc   0  -0.01570  1.48700   0 -0.00785  1.48700
ghn   0  -0.01570  0.60000   0 -0.00785  0.60000
gna   0  -0.17000  1.82400   0 -0.08500  1.82400
gnb   0  -0.17000  1.82400   0 -0.08500  1.82400
gnc   0  -0.17000  1.82400   0 -0.08500  1.82400
gnd   0  -0.17000  1.82400   0 -0.08500  1.82400
gos   0  -0.17000  1.68370   0 -0.08500  1.68370
```
